# Synthesis and Evaluation of Eleven Novel Renewable Plasticizers for Polylactic Acid (PLA): Linking Molecular Structure to Performance

**DOI:** 10.3390/polym18172070

**Published:** 2026-08-26

**Authors:** Ferry Oomen, Marc Crockatt, Eric Mattheussens, Ivan Bakker, Michał Pstrowski, Maddalena Logrieco, Moctar Coulibaly, Han van Kasteren, Sandra Corderí Gándara

**Affiliations:** 1 Centre of Expertise Materials and Energy Transition (MNEXT), Lovensdijkstraat 63, 4818 AJ Breda, The Netherlands; f.oomen@avans.nl (F.O.); esgm.mattheussens@avans.nl (E.M.); me.logrieco@avans.nl (M.L.); m.coulibaly@avans.nl (M.C.); 2 Department of Sustainable Process and Energy Systems, TNO, Kessler Park 1, 2288 GH Rijswijk, The Netherlands; marc.crockatt@tno.nl (M.C.); ivan.bakker@tno.nl (I.B.); michal.pstrowski@tno.nl (M.P.); 3 Materials and Chemistry Unit (MatCh), Flemish Institute for Technological Research (VITO N.V.), Boeretang 200, 2400 Mol, Belgium

**Keywords:** bio-plasticizers, renewable feedstocks, polymer additives, polylactic acid, Diels–Alder reaction, furan, Hansen Solubility Parameters

## Abstract

The development of bio-based plasticizers is increasingly important due to regulatory restrictions on several phthalates, growing concerns over plasticizer migration and toxicity, and the demand for renewable materials. In this work, eleven novel furfural-derived bio-based plasticizers were synthesized and evaluated in polylactic acid (PLA) at 15 wt% loading via melt compounding. Their influence on the thermomechanical properties of PLA was investigated, enabling the establishment of structure–property relationships. Hansen Solubility Parameters (HSPs) were used to predict plasticizer–PLA compatibility and correlate theoretical predictions with experimental performance. All developed bio-plasticizers exhibited onset degradation temperatures exceeding 225 °C, ensuring suitability for melt processing with PLA. Increasing the alkyl chain length in the diester bio-plasticizers reduced plasticization efficiency, consistent with lower predicted compatibility based on HSP analysis. The most promising candidates, namely 7-oxabicyclo[2.2.1]heptane-2,3-dicarboxylic acid 2,3-dihexyl ester (F/MA-C6), 7-oxabicyclo[2.2.1]heptane-2,3-dicarboxylic acid 2,3-dioctyl ester (F/MA-C8), and 1,1′-[oxybis(1-methyl-2,1-ethanediyl)]bistetrahydrofuroate (Bis-THF), substantially enhanced polymer chain mobility, reducing the glass transition temperature to approximately 28–35 °C compared to 60 °C for neat PLA. These bio-plasticizers also demonstrated outstanding plasticizing efficiency, achieving elongations at break exceeding 245%, compared with 4.5% for neat PLA. These results demonstrate that furfural-derived plasticizers are promising sustainable alternatives to conventional fossil-based plasticizers for flexible PLA applications.

## 1. Introduction

Plasticizers are short-to-mid-chain additives used to tailor polymer properties for specific applications. A plasticizer is a substance or material incorporated in a material (usually a plastic or elastomer) to increase its flexibility, workability, or distensibility [1]. When introduced into a polymer matrix, plasticizers weaken intermolecular forces, resulting in a softer, less brittle, and more easily processed material [2]. Plasticizer selection is governed by polymer compatibility, targeted thermal and mechanical properties, environmental and chemical stability, toxicity, cost, and processability [3]. These additives are used in a range of polymers, including polyvinylics, acrylics, cellulose derivatives, polyamides, and polylactic acid (PLA). Among these, polyvinyl chloride (PVC) accounts for 80–90% of the global plasticizer consumption, corresponding to a market of 8.6 million tons in 2023, for applications such as wires and cables, coatings, flooring, and packaging [4,5]. By the 1920s, phthalic acid esters had gained widespread application as plasticizers and later became the dominant class of plasticizers, representing approximately 80% of global plasticizer production [6,7].

Plasticizers are commonly classified by their function and interaction with polymers. Functionally, primary plasticizers are highly compatible with the polymer and used as the main plasticizing agent, whereas secondary plasticizers are employed in smaller quantities to fine-tune material properties or enhance cost-effectiveness. Through interactions with the polymer, external plasticizers are physically mixed and may migrate, whereas internal plasticizers are chemically bonded to the polymer and are retained within the polymer structure [8]. External plasticizers are widely used due to their ease of incorporation, enabling straightforward adjustment of material properties while maintaining a cost-effective formulation strategy [9]. Among external plasticizers, phthalates are particularly concerning, as they can migrate out of polymers over time, and some are associated with serious health and environmental concerns [10,11]. Since 2020, the EU has restricted the use of the bis(2-ethylhexyl) phthalate (DEHP), dibutyl phthalate (DBP), benzyl butyl phthalate (BBP), and isobutyl phthalate (DIBP) across many products, including children’s toys, flooring, coated fabrics, and many other consumer goods, if their combined concentration exceeds 0.1 wt% of the plasticized material [12]. Growing health and environmental concerns over fossil-derived phthalate plasticizers, coupled with tightening EU regulations, are accelerating the transition toward sustainable renewable plasticizer alternatives [2,13]. As a result, the European bio-based plasticizers market is projected to grow steadily over the next decade (CAGR 8.2%, 2024–2031 [14]).

Recent review studies have summarized the development and application of bio-based plasticizers [2,15,16,17]. Several of these bio-based plasticizers are commercially available, among which epoxidized vegetable oils represent one of the most extensively utilized classes. In addition, glycerol, succinates, citrates, cardanol, and water have been explored as alternatives to conventional plasticizers [2,17]. Nevertheless, in most cases, further tailoring of the functionality to improve polymer compatibility, along with additional efficiency improvements and cost reductions, are still required for broader implementation. A novel class of bio-plasticizers, the bicyclic oxa-bridged diesters synthesized using the furan-derived 2-methylfuran, maleic anhydride, and 2-ethylhexanol, is particularly interesting because of the structural similarities to phthalates, with demonstrations of promising performances in PVC [18].

The majority of bio-based plasticizers are used in PVC, while only a limited fraction is applied to bio-based polymers, such as polylactic acid (PLA) [17]. As the leading polymer in the bio-based and biodegradable plastics segment, PLA combines environmental compatibility with stiffness and transparency. The incorporation of bio-based plasticizers enhances its mechanical and processing properties, thereby expanding its use in sustainable material applications [15,19]. Acetyl tributyl citrate, tributyl citrate, epoxidized soybean oil (ESO), and polyethylene glycols (PEGs) are the most commonly used bio-based plasticizers for PLA [15,20,21]. For example, PLA/PEG-400 blends exhibited a reduction in glass transition and cold crystallization temperatures with an increase in PEG content up to 20 wt%. However, phase separation occurred when the plasticizer content was increased to 30 wt% [21]. Studies of PLA/PEG-4000 and PLA/ESO blends at 15 wt% bio-plasticizer loading raised the elongation at break to 99% and 6%, respectively [22].

Despite numerous sustainable plasticizers being proposed also for PLA, new bio-based alternatives are needed, as most existing options face scalability or performance limitations. To address these gaps, we developed and evaluated 11 unique bio-based plasticizers for PLA, thereby expanding the range of sustainable and viable alternatives. Bio-based polymers like PLA require plasticizers with tailored compatibility, for which Hansen Solubility Parameters (HSPs) serve as a useful predictive tool linking polymer/plasticizer compatibility to molecular structures [23]. Plasticizers with Hansen parameters similar to those of the polymer are generally more compatible, leading to improved miscibility, reduced phase separation, and enhanced long-term performance.

The objective of this study was to synthesize and functionally appraise eleven bio-derivatives of two types of plasticizers. Nine novel bicyclic oxa-bridged diester bio-plasticizer derivatives, with structural similarities to conventional phthalates, and two new di(propylene glycol)-derived bio-plasticizers, structurally similar to di(propylene glycol) dibenzoate (DPGDB), were produced from renewable feedstocks. These bio-plasticizers were characterized by proton nuclear magnetic resonance (^1^H NMR) spectroscopy, gas chromatography–mass spectrometry (GC-MS), and their thermal properties. Their technical performance was evaluated in PLA through thermomechanical characterization, and their structure–property relationships were established. Furthermore, Hansen Solubility Parameters were used to assess the theoretical compatibility of the new bio-plasticizers with PLA and to correlate these predictions with their experimental performance. Conventional plasticizers (diisononyl phthalate (DINP), PEG-400, and DPGDB) were also evaluated for comparison with the bio-based alternatives. The development of novel bio-based plasticizers structurally analogous to conventional plasticizers may enable improved compatibility with PLA while maintaining plasticization efficiency and contributing to the transition toward fully bio-based polymer systems.

## 2. Materials and Methods

### 2.1. Materials

Eleven unique bio-based plasticizers were synthesized by TNO, the Netherlands Organization for Applied Scientific Research, using renewable resources. The chemicals employed in the synthesis and characterization of the bio-plasticizers, along with their purities and suppliers, are detailed as follows: furan (≥99.0%, Sigma-Aldrich, Amsterdam, The Netherlands), 2-methylfuran (≥98.0%, TCI), 2,5-dimethylfuran (99%, Sigma-Aldrich), maleic anhydride (99%, Sigma-Aldrich), methyl tert-butyl ether (MTBE) (99%, Thermo Fisher Scientific), palladium on activated charcoal (10% Pd basis, Sigma-Aldrich), hydrogen (≥99.999%, Linde Gas, Dublin, Ireland), tetrahydrofuran (THF) (≥99%, VWR Chemicals, Amsterdam, The Netherlands), sulfuric acid (≥95%, VWR Chemicals), 1-butanol (99.8%, Sigma-Aldrich), 1-hexanol (≥98%, Merck, Darmstadt, Germany), 1-octanol (≥99%, Sigma-Aldrich), 1-dodecanol (98%, Sigma-Aldrich), 1-hexadecanol (≥99%, Sigma-Aldrich), sodium bicarbonate (≥99.0%, Merck), sodium hydroxide (≥98%, Sigma-Aldrich), sodium chloride (≥99.0%, Sigma-Aldrich), activated carbon (NORIT^®^ RX3 EXTRA, Sigma-Aldrich), magnesium sulfate (≥98.0%, VWR Chemicals), toluene (≥99.7%, Biosolve, Valkenswaard, The Netherlands), furfuryl alcohol (≥98%, Sigma-Aldrich), potassium hydroxide (≥84%, Merck), Aliquat-336 (88.2–93.0%, Sigma-Aldrich), butyl iodide (99%, Sigma-Aldrich), octyl bromide (99%, Sigma-Aldrich), dodecyl bromide (97%, Sigma-Aldrich), sodium sulfate (≥99%, Sigma-Aldrich), tetrahydro-2-furoic acid (≥97%, Thermo Scientific Chemicals), 2-furoic acid (98%, Sigma-Aldrich), thionyl chloride (≥99%, Sigma-Aldrich), 2-methyltetrahydrofuran (≥99.5%, Sigma-Aldrich), dipropylene glycol (≥97.0%, Sigma-Aldrich), triethylamine (≥99.5%, Sigma-Aldrich), hydrochloric acid (37%, Sigma-Aldrich), n-heptane (≥95%, Biosolve), ethyl acetate (≥99.5%, Biosolve), deuterated chloroform [CDCl_3_] (99.8% D, VWR Chemicals), deuterated dimethyl sulfoxide [DMSO-d_6_] (≥99.96%, Sigma-Aldrich), nitromethane (≥99.0%, Sigma-Aldrich), dimethyl maleate (99.0%, Apollo Scientific, Manchester, UK), dimethyl fumarate (≥99.0%, Thermo Fisher Scientific), dimethyl carbonate (≥99.0%, Sigma-Aldrich), ethylene carbonate (≥99.0%, Thermo Fisher Scientific), dichloromethane (≥99.9%, Biosolve), methanol (≥99.98%, Biosolve), and helium (≥99.999%, Linde Gas).

**Figure 1:**
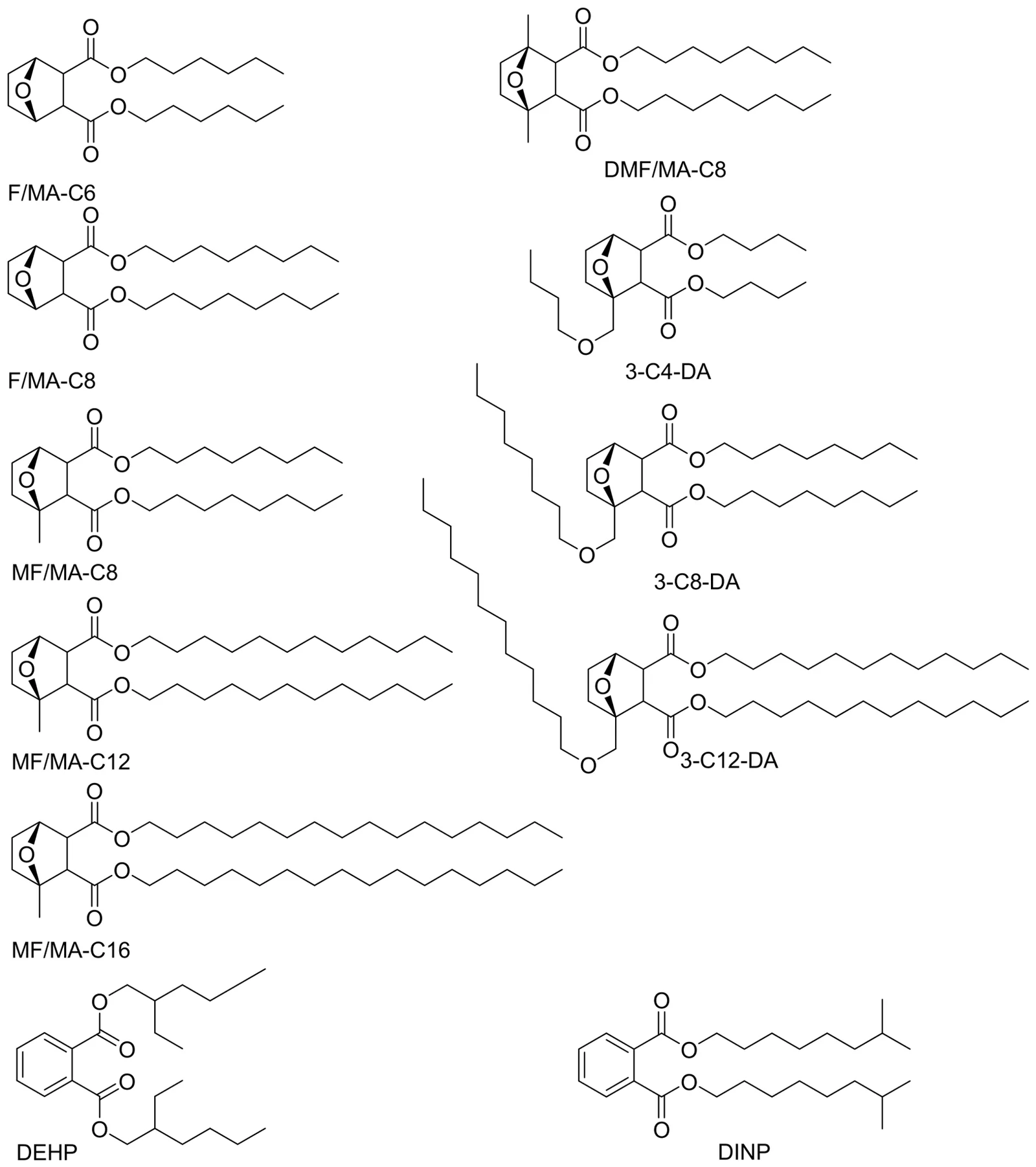
Chemical structures of the developed bio-based bicyclic oxa-bridged diester plasticizers (F/MA-C6, F/MA-C8, MF/MA-C8, MF/MA-C12, MF/MA-C16, DMF/MA-C8, 3-C4-DA, 3-C8-DA, and 3-C12-DA) and common phthalate plasticizers (DEHP and DINP).

**Figure 2:**
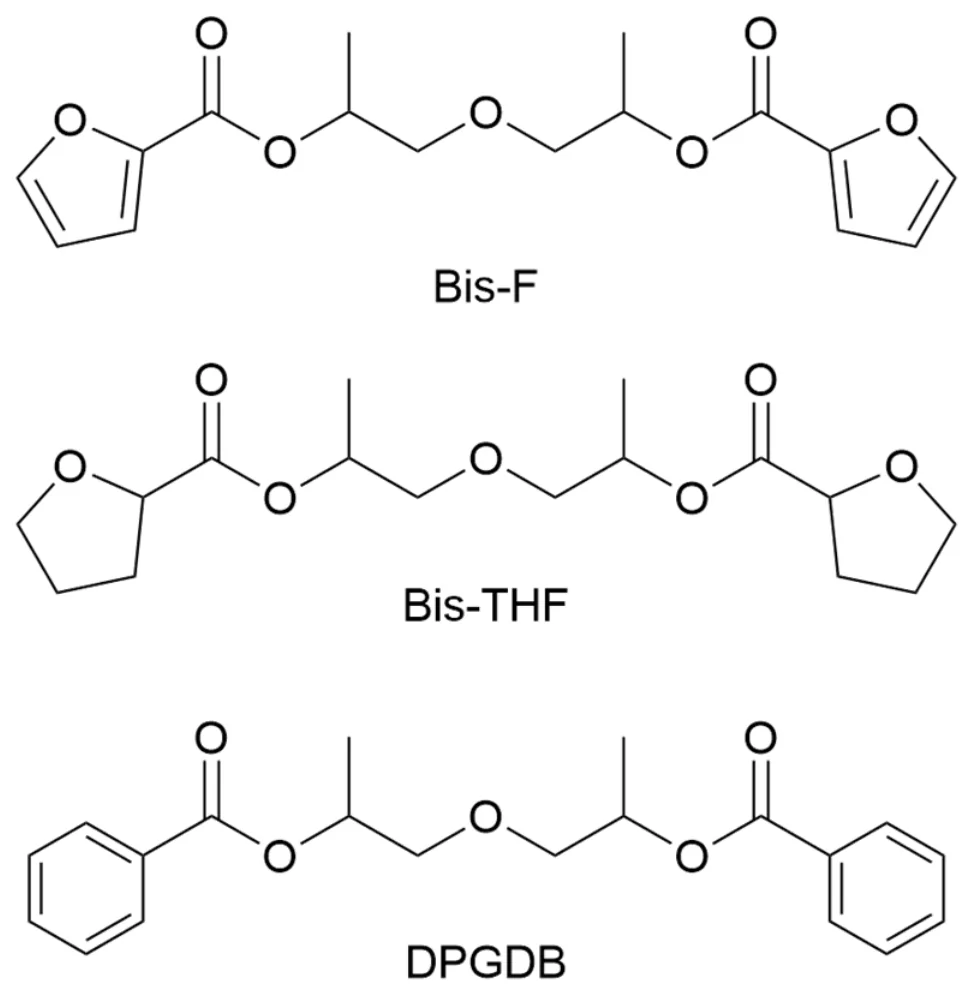
Chemical structures of the developed bio-based di(propylene glycol) plasticizer derivatives Bis-F and Bis-THF, with di(propylene glycol) dibenzoate (DPGDB) as a reference.

The chemical structures of the novel bio-plasticizers can be found in Figure 1 and Figure 2, and their basic descriptions are in Table 1. Polyethylene glycol 400 (PEG-400) (~99%, Acros organics, Geel, Belgium). Di(propylene glycol) dibenzoate (DPGDB) (78.2%, Sigma-Aldrich) and diisononyl phthalate (DINP) (≥99.5%, Sigma-Aldrich) were purchased from Merck. The bio-polymer polylactic acid (PLA), Ingeo™ Biopolymer 3052D, of injection molding grade was supplied by NatureWorks LLC, Naarden, The Netherlands.

### 2.2. Bio-Plasticizer Synthesis Procedures

#### 2.2.1. Synthesis of the F/MA-C6, F/MA-C8, MF/MA-C8, MF/MA-C12, MF/MA-C16, and DMF/MA-C8 Bio-Plasticizers

**Scheme 1:**
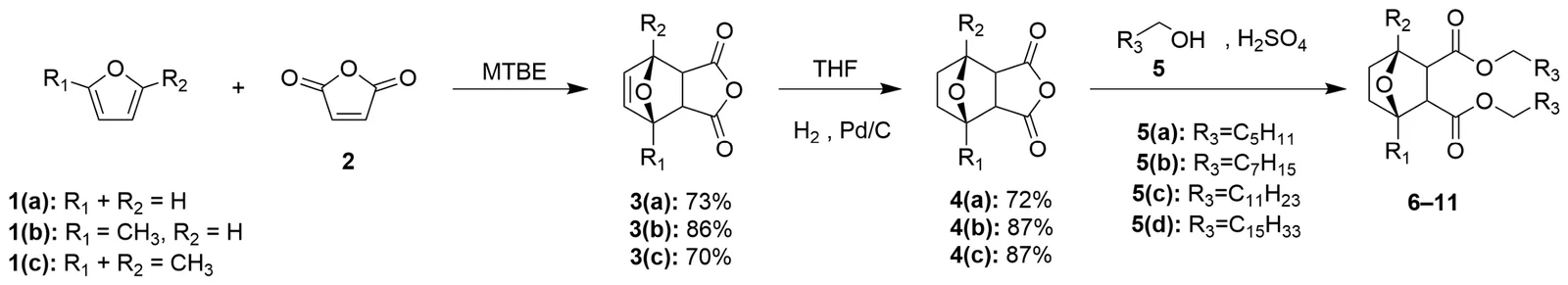
Synthesis of the bicyclic oxa-bridged diester bio-plasticizers F/MA-C6, F/MA-C8, MF/MA-C8, MF/MA-C12, MF/MA-C16, and DMF/MA-C8 (**6–11**, respectively).

The synthesis pathways of the bio-based bicyclic oxa-bridged diester plasticizers F/MA-C6, F/MA-C8, MF/MA-C8, MF/MA-C12, MF/MA-C16, and DMF/MA-C8 (**6–11**, respectively) are presented in Scheme 1. These bio-plasticizers were synthesized in accordance with a previously reported procedure [24]. The process involves the synthesis of the corresponding unsaturated anhydrides (**3a**, **3b**, and **3c**) via Diels–Alder reactions of bio-furfural derivatives (**1a**, **1b**, and **1c**) with maleic anhydride (**2**), followed by a hydrogenation step to produce **4a**, **4b**, and **4c** intermediates and a ring opening and esterification step with the alcohols **5a–d** to yield the bio-plasticizers **6–11**. ^1^H NMR spectroscopy and GC-MS were used to analyze the intermediate reaction products and final bio-plasticizers.

- Synthesis of unsaturated anhydrides (**3a–c**) via Diels–Alder reactions

Maleic anhydride (**2**) was dissolved in methyl tert-butyl ether (MTBE) at ambient temperature or heated to facilitate complete dissolution. Depending on the product (**3a**, **3b**, or **3c**), the internal temperature was maintained below 30–50 °C, and the appropriate furfural derivative (furan (**1a**), 2-methylfuran (**1b**), or 2,5-dimethylfuran (**1c**)) was then introduced dropwise. After this addition, the mixture was briefly stirred and cooled to 7–10 °C to induce crystallization. The resulting solid was isolated by filtration, washed with a minimal amount of ice-cold MTBE, and dried under a vacuum at 20 °C. The reactions yielded the following products: 7-oxabicyclo[2.2.1]hept-5-ene-2,3-dicarboxylic anhydride (**3a**) and its 1-methyl (**3b**) and 1,4-dimethyl derivatives (**3c**).

- Synthesis of hydrogenated anhydrides (**4a–c**)

An autoclave was charged with the corresponding unsaturated anhydride (**3a–c**) and tetrahydrofuran (THF), followed by 10% Pd/C under a nitrogen atmosphere, according to the method proposed by S. Thiyagarajan et al. [25]. The reactor was sealed, flushed twice with nitrogen to a pressure of 15 bar, and charged with hydrogen up to 80 bar. Stirring was started, and hydrogen was recharged to 80 bar when the pressure dropped below 50 bar. The mixture was stirred until the internal temperature fell below 25 °C, then the hydrogen pressure was released, and the reactor was flushed twice with nitrogen. The mixture was filtered to remove the catalyst, concentrated via evaporation, and, upon cooling, seeded with the product to induce crystallization. The solid was collected by filtration, washed with a minimal amount of ice-cold THF, and dried under a vacuum at 20 °C before purification by distillation, yielding 7-oxabicyclo[2.2.1]heptane-2,3-dicarboxylic anhydride (**4a**), 1-methyl-7-oxabicyclo[2.2.1]heptane-2,3-dicarboxylic anhydride (**4b**), and 1,4-dimethyl-7-oxabicyclo[2.2.1]heptane-2,3-dicarboxylic anhydride (**4c**).

- Synthesis of bio-plasticizers **6–11** via ring opening and esterification

A reactor was charged with the selected hydrogenated anhydride (**4a**, **4b** or **4c**), the corresponding alcohol (1-hexanol (**5a**), 1-octanol (**5b**), 1-dodecanol (**5c**), and 1-hexadecanol (**5d**), and concentrated sulfuric acid, utilizing a method adapted from C. Plass et al. [18]. The reaction mixture was heated to 90 °C while stirring and held at this temperature for 18–60 h. The reaction mixture was cooled to room temperature, then ethyl acetate was added, and the mixture was washed with a saturated sodium bicarbonate solution. This process yielded F/MA-C6 (**6**) and F/MA-C8 (**7**). Additional washing steps were required to produce the MF/MA-C8 (**8**), MF/MA-C12 (**9**), and DMF/MA-C8 (**11**) bio-plasticizers. In this case, the reaction mixture was washed twice with 1 M sodium hydroxide, followed by a final wash with saturated brine. The organic phase was separated, stirred with activated carbon RX3 for 1 h at ambient temperature, filtered, dried over anhydrous magnesium sulfate, filtered, and then concentrated under reduced pressure to yield the bio-plasticizers (**8**, **9**, **11**). An alternative approach was required to synthesize the MF/MA-C16 bio-plasticizer (**10**). A reactor was charged with **4b**, 1-hexadecanol (**5d**), concentrated sulfuric acid, and toluene. The reaction mixture was heated to reflux while stirring under Dean–Stark conditions and held for 12 h. Subsequently, the reaction mixture was cooled slightly and washed with saturated sodium bicarbonate solution. The organic phase was separated, dried over magnesium sulfate, filtered, and then slowly cooled to 0 °C while being stirred continuously. The formed solid was isolated by filtration, washed with a minimal amount of ice-cold toluene, and dried in a vacuum oven to yield the MF/MA-C16 bio-plasticizer (**10**).

#### 2.2.2. Synthesis of the 3-C4-DA, 3-C8-DA, and 3-C12-DA Bio-Plasticizers

**Scheme 2:**
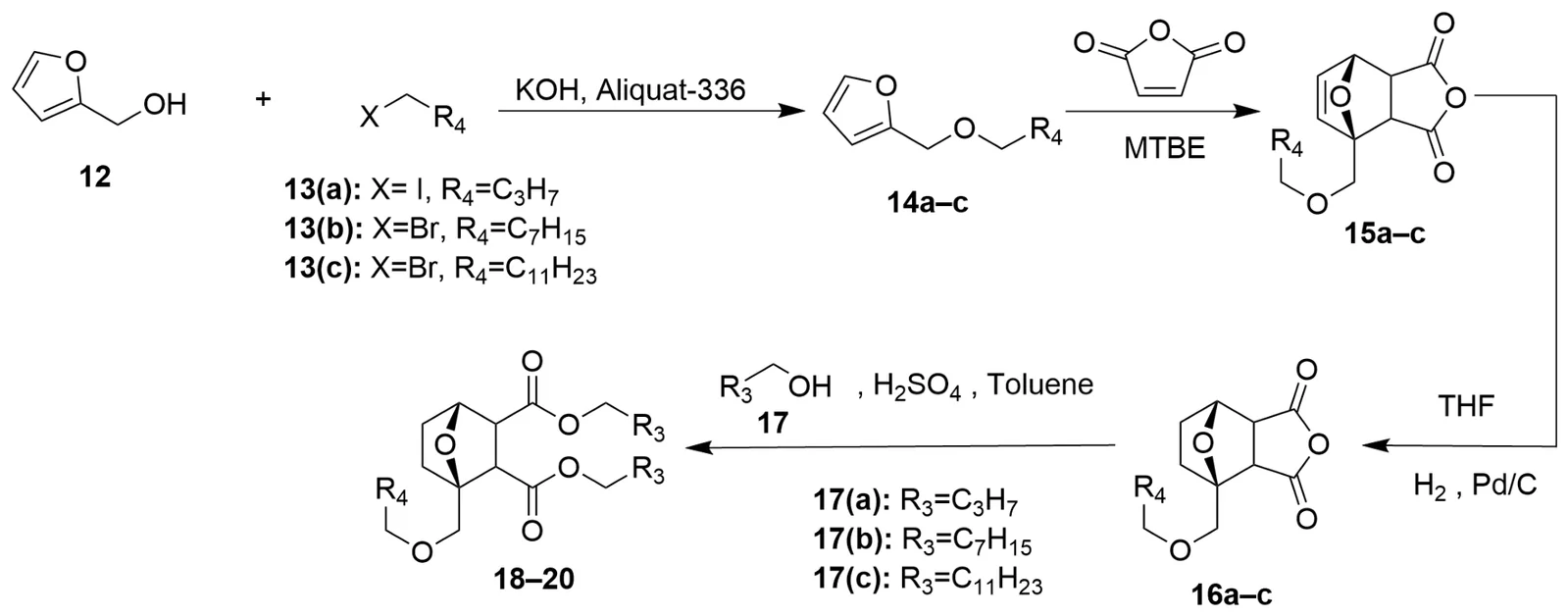
Synthesis of bicyclic oxa-bridged diester 3-C4-DA, 3-C8-DA, and 3-C12-DA bio-plasticizers (**18**, **19**, and **20**, respectively).

The bio-based bicyclic oxa-bridged diester plasticizers 3-C4-DA, 3-C8-DA, and 3-C12-DA (**18**, **19**, and **20**, respectively) were synthesized from bio-based furfuryl alcohol (**12**) via the formation of furfuryl ether derivatives (**14a–c**)following the previously described procedure [26]. These intermediates were reacted with maleic anhydride to yield the corresponding unsaturated anhydrides (**15a–c**), which were subsequently hydrogenated and esterified with the alcohols **17a**, **17b**, or **17c** to obtain the target bio-plasticizers (**18–20**). These synthesis steps are shown in Scheme 2. The intermediate reaction products and the final bio-plasticizers were analyzed by ^1^H-NMR spectroscopy and GC-MS.

- Synthesis of furfuryl ether derivatives (**14a–c**)

A reactor was charged with potassium hydroxide and Aliquat-336, and the mixture was stirred at 20 °C. Furfuryl alcohol (**12**) was added to the reactor, followed by the corresponding addition of butyl iodide (**13a**), octyl bromide (**13b**), or dodecyl bromide (**13c**). The reaction mixture was heated to 80 °C and maintained at this temperature for 120 min, after which it was cooled to room temperature. Afterwards, the mixture was partitioned between water and MTBE. The organic phase was dried over sodium sulfate, filtered, and then concentrated to yield a liquid. Vacuum distillation was used to purify and yield butyl-furfuryl ether (**14a**), octyl-furfuryl ether (**14b**) or dodecyl-furfuryl ether (**14c**).

- Synthesis of unsaturated anhydrides (**15a–c**) via Diels–Alder reactions

The unsaturated anhydrides 4-(butoxymethyl)-3a,4,7,7a-tetrahydro-4,7-epoxyisobenzofuran-1,3-dione (**15a**), 4-(octoxymethyl)-3a,4,7,7a-tetrahydro-4,7-epoxyisobenzofuran-1,3-dione (**15b**), and 4-(dodecoxymethyl)-3a,4,7,7a-tetrahydro-4,7-epoxyisobenzofuran-1,3-dione (**15c**) were synthesized via Diels–Alder reactions with maleic anhydride following a procedure analogous to that used for the synthesis of **3a–c**, using the corresponding furfuryl ether derivatives (**14a–c**) as substrates.

- Synthesis of hydrogenated anhydrides (**16a–c**)

The synthesis of the hydrogenated anhydrides (**16a–c**) from the corresponding unsaturated anhydrides (**15a–c**) was carried out under conditions analogous to those used for the preparation of **4a–c**. In this case, the reaction mixture was filtered to remove the palladium catalyst, and the filtrate was concentrated under reduced pressure to yield a crude liquid product. Purification was performed by flash column chromatography using a gradient of 0–40% ethyl acetate in *n*-heptane. Fractions containing the desired product were combined and concentrated under reduced pressure to yield the corresponding saturated dione products: 4-(butoxymethyl)hexahydro-4,7-epoxyisobenzofuran-1,3-dione (**16a**), 4-(octoxymethyl)hexahydro-4,7-epoxyisobenzofuran-1,3-dione (**16b**), and 4-(dodecoxymethyl)hexahydro-4,7-epoxyisobenzofuran-1,3-dione (**16c**).

- Synthesis of bio-plasticizers **18–20** via ring opening and esterification

A reactor was charged with one of the three isobenzofuran-1,3-diones **16a**, **16b** or **16c**—together with the corresponding alcohol (1-butanol (**17a**), 1-octanol (**17b**), or 1-dodecanol (**17c**)), concentrated sulfuric acid, and toluene. The reaction mixture was heated to reflux and stirred under Dean–Stark conditions for 12 h. Once cooled to room temperature, the mixture was successively washed with a saturated aqueous sodium bicarbonate solution, twice with 1 M aqueous sodium hydroxide, and then followed by a final wash with a saturated brine solution. The organic layer was separated and treated with activated carbon (RX3) at 100 °C for one hour. The suspension was then cooled to 20 °C, filtered, dried over anhydrous magnesium sulfate, filtered again, and then concentrated under reduced pressure to yield a crude liquid product. The residue was purified by flash chromatography (0–20% ethyl acetate in n-heptane). The appropriate fractions were then combined and concentrated under reduced pressure to yield the final products of 3-C4-DA (**18**), 3-C8-DA (**19**), and 3-C12-DA (**20**).

#### 2.2.3. Synthesis of the Bio-Based Di(propylene Glycol) Plasticizer Derivatives Bis-F and Bis-THF

**Scheme 3:**
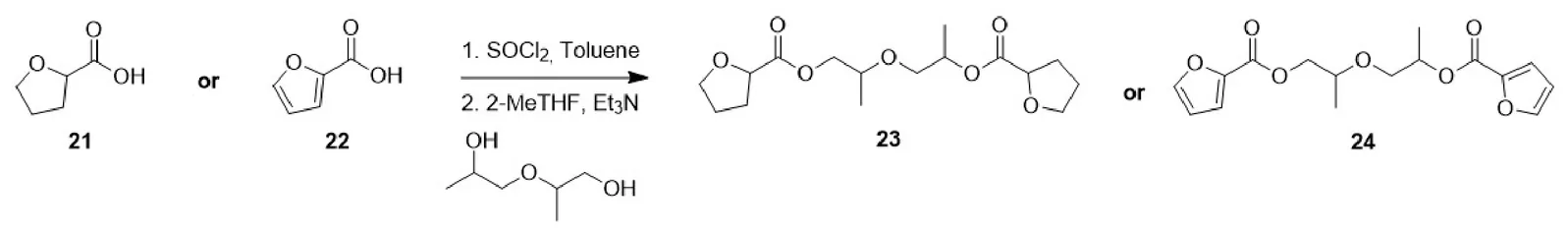
Synthesis of the bio-based di(propylene glycol) plasticizer derivatives Bis-F (**23**) and Bis-THF (**24**).

Scheme 3 shows the synthetic route for the bio-based di(propylene glycol) plasticizer derivatives Bis-F (**23**) and Bis-THF (**24**), which were prepared via esterification of tetrahydro-2-furoic acid (**21**) and 2-furoic acid (**22**) with dipropylene glycol, respectively. Both 2-furoic acid and its hydrogenated derivative, tetrahydro-2-furoic acid, are produced from biomass-derived furfural.

A reactor was charged with tetrahydro-2-furoic acid (**21**) or 2-furoic acid (**22**), along with toluene. The reaction mixture was maintained at 20 °C, and thionyl chloride was added dropwise over approximately 60 min. Afterwards, the reaction mixture was heated to 80 °C and stirred for 2 h, following which it was then cooled to 20 °C. The solvent and excess thionyl chloride were then removed under reduced pressure to yield crude tetrahydro-2-furoyl chloride or crude 2-furoyl chloride. Following that, the reactor was charged with dipropylene glycol, 2-methyltetrahydrofuran and triethylamine. Stirring was started at 20 °C, and a solution of 2-furoyl chloride or tetrahydro-2-furoyl chloride in 2-methyltetrahydrofuran was added dropwise over approximately 60 min. The reaction mixture was then stirred at 20 °C for 4 h. Subsequently, the mixture was successively washed with 1 M aqueous hydrochloric acid (twice), 1 M aqueous sodium hydroxide (twice), water (once), and saturated brine (once). Thereafter, the organic phase was dried over anhydrous sodium sulfate and then treated with activated carbon (RX3) at reflux for 4 h. The mixture was allowed to cool to 20 °C, whereafter it was filtered and concentrated under reduced pressure to yield a crude liquid. This liquid was further purified using flash chromatography (10% to 50% ethyl acetate in n-heptane). The product-containing fractions were collected and concentrated under reduced pressure to yield the bio-plasticizers Bis-F (**23**) or Bis-THF (**24**). ^1^H-NMR spectroscopy and GC-MS were used to analyze the purified bio-plasticizers.

### 2.3. Characterization of (Bio)-Plasticizers

Proton nuclear magnetic resonance (^1^H NMR) spectra were recorded using either a 400 MHz Bruker spectrometer (Bruker, Billerica, MA, USA) or a60 MHz Magritek Spinsolve Benchtop NMR (Magritek, Wellington, New Zealand). Samples (10–40 mg) were dissolved in an appropriate deuterated solvent (e.g., CDCl_3_ or DMSO-d_6_). For quantitative ^1^H NMR analysis, a known amount of an internal standard (nitromethane, dimethyl maleate, dimethyl fumarate, dimethyl carbonate, ethylene carbonate) was added to the solution. Chemical shifts are reported in parts per million (ppm) relative to the residual solvent peak.

Gas chromatography–mass spectrometry (GC-MS) analysis was performed on a Thermo-Fisher Trace 1310 gas chromatograph coupled with an ISQ series mass spectrometer (Thermo Fisher Scientific, Waltham, MA, USA). The system was equipped with a Restek RTX-200 capillary column (Restek Corporation, Bellefonte, PA, USA). Samples were prepared by dissolving approximately 0.1 mg of material in an appropriate solvent (dichloromethane, methanol) and filtering the mixture through a 0.45 µm PTFE syringe filter. An injection volume of 0.5 µL was used in split mode (split ratio 17:1, split flow 20.0 mL/min, purge flow 5.0 mL/min) with an injector temperature of 260 °C. Helium was used as the carrier gas at a constant flow rate of 1.2 mL/min. The oven temperature program started at 40 °C (held for 1 min), ramped at 15 °C/min to 300 °C, and finished with a 3 min hold at 300 °C (total runtime 21.3 min). The MS transfer line and ion source temperatures were maintained at 300 °C. Data were acquired in the mass range of 33–500 *m*/*z*, with a dwell time of 0.028 s.

Thermogravimetric analysis (TGA) was performed on a TGA 550 (TA Instruments, New Castle, DE, USA). Approximately (5–20 mg) of each sample was placed in a pre-cleaned and pre-tared platinum pan (100 µL). The measurements were carried out in an inert nitrogen atmosphere (N_2_ flow 60 mL/min), with a heating rate of 20 °C/min from ambient temperature to 750 °C. Each sample was measured in duplicate to ensure reproducibility. The degradation onset temperature (T_d_-onset) was determined from the TGA curves using the tangent-intersection method (extrapolation of the baseline and the steepest slope of the leading weight-loss step). The maximum degradation temperature (T_d_-max) was taken as the temperature at the peak of the derivative thermogravimetric curve.

### 2.4. Incorporation of (Bio)-Plasticizers in PLA via Melt Compounding

The (bio)-plasticizers were incorporated into PLA using a HAAKE™ MiniLab 3 Micro Compounder (Thermo Fisher Scientific, Waltham, MA, USA) equipped with a conical twin-screw configuration, a pneumatic feeding system and a recirculation system. Prior to processing, PLA granules were dried in a convection oven (Memmert UN55, Memmert GmbH + Co. KG, Schwabach, Germany) at 50 °C for at least 24 h to minimize hydrolytic degradation. Compounding was conducted on a 5 g scale using PLA with a 15 wt% loading of (bio)-plasticizer at 190 °C for 60 s, during which the melt was continuously recirculated. The screws had a diameter of 5/14 mm, a total length of 109.4 mm, and the rotation speed was maintained at 100 rpm. After compounding, the melt was discharged via a heated cylinder for injection molding.

### 2.5. Injection Molding of Specimens

Injection molding with a HAAKE™ MiniJet Pro Piston Injection Molding System (Thermo Fisher Scientific, Waltham, MA, USA) was used to produce tensile bars and disks in accordance with ISO 527–2 [27], Type 5A and ISO 6721–10 [28], respectively. The material from the compounder was collected with the heated cylinder at 190 °C and injected into the pre-heated mold at 35 °C with a pressure of 570 bar over 2 s, followed by an after-pressure of 470 bar for an additional 4 s. The produced samples were stored in sealed plastic bags before subjecting them to thermal and mechanical characterization.

### 2.6. Characterization of Produced PLA–(Bio)-Plasticizer Specimens

DSC measurements were carried out on the different PLA–(bio)-plasticizer blends. Samples (5–10 mg) from the injection-molded tensile bars were measured within the temperature range of −50 °C and 200 °C. The DSC measurement consisted of a heat–cool–heat cycle with heating at 10 °C/min and cooling at 5 °C/min, while the nitrogen flow was 50 mL/min. All samples were measured in duplicate, and the glass transition temperature (T_g_) and melting temperature (T_m_) were determined.

Mechanical testing (tensile testing) was conducted to determine the mechanical properties of PLA and the specimens incorporating the (bio-based) plasticizers. The tests were performed using a Shimadzu AGS-X universal testing machine (Shimadzu Corporation, Kyoto, Japan) equipped with a 1 kN load cell. Each tensile bar was initially subjected to a crosshead speed of 5 mm/min until a measurable load was detected, after which the speed was increased to 20 mm/min and maintained until specimen failure. The Young’s modulus, stress at break, and elongation at break were calculated from the obtained stress–strain curves. To ensure statistical reliability, a minimum of six replicates were tested for each sample formulation.

Rheology was carried out on PLA–(bio)-plasticizer blends using the Core Rheometer (CR), HR20, from TA Instruments (New Castle, DE, USA). The complex viscosity (Pa·s), storage modulus (G′, Pa), and loss modulus (G″, Pa) were measured on injection-molded disks in accordance with ISO 6721–10 [28]. Oscillatory rheology was performed at 190 °C using a logarithmic frequency sweep from 628 to 0.1 rad s^−1^ (five points per decade, 20 measurements in total), under direct strain control with a 1% strain amplitude. Continuous oscillation was applied with automatic instrument control enabled and the inherent set-point function disabled. The samples were equilibrated (soaked) for 180 s, and the measurements started once the target temperature was reached. The target temperature was set at 190 °C to determine the rheological effects during the processing of the material.

## 3. Results and Discussion

### 3.1. Bio-Plasticizer Synthesis Outcomes

Eleven furfural-derived novel bio-plasticizers were synthesized. To the best of our knowledge and based on a literature search performed up to May 2026, these compounds have not previously been reported as plasticizers. The reaction yields and purities determined by ^1^H NMR spectroscopy and GC-MS of the reaction intermediates and purified bio-plasticizers are depicted in Table 2. The ^1^H NMR spectra and mass spectra can be found in the Supplementary Materials (Figures S1–S18).

Bio-based plasticizers 6–11 were synthesized via a three-step sequence that started from renewable furanic substrates. Furfural/5-(hydroxymethyl)furfural-derived furanics (furan, 2-methylfuran, and 2,5-dimethylfuran) were reacted with maleic anhydride, a dienophile that can also be sourced from biomass (e.g., furfural, hydroxymethyl furfural, levulinic acid, n-butanol and bio-derived diols), in a Diels–Alder cycloaddition employing a reactive crystallization approach. This afforded adducts **3a–c** in 73%, 86%, and 70% yields, respectively, consistent with previously reported values for similar materials [24]. Subsequent catalytic hydrogenation led to products **4a–c** in 72%, 87%, and 87% yields, respectively, with purities exceeding 99% by ^1^H NMR, in line with the literature’s precedents [25]. Finally, bio-plasticizers **6–11** were obtained after an acid-catalyzed esterification step with fatty alcohols (C6, C8, C12, and C16) in moderate-to-good yields (42–86%) and a high purity (≥98.0% by ^1^H NMR; ≥96.0% by GC-MS), which was also in line with previously reported results [18].

Bio-plasticizers **18–20** were synthesized via a related route incorporating an initial etherification step. Bio-based furfuryl alcohol was alkylated with fatty alkyl halides (C4, C8, and C12), affording ethers **14a–c** in high yields (81–106%) following distillation, with low-level residual alcohol contents. The resulting ethers underwent Diels–Alder cycloadditions with maleic anhydride under the same reactive crystallization conditions as previously described, yielding adducts **15a–c** in 53–82%. While slightly more variable than the non-etherified analogs, these yields remain within the range reported for substituted furan systems [26]. Subsequent hydrogenation (Pd/C) afforded products **16a–c** in 53–79% yields, with excellent purity (>99%), after flash chromatography. Final esterification with fatty alcohols (C4, C8 and C12) provided plasticizers **18–20** in moderate yields (62–77%) but with high purity (≥95.0% by ^1^H NMR; ≥98.0% by GC-MS). Bio-plasticizers **23** and **24** were prepared via a shorter route that began with furfural-derived acids (2-furoic acid and tetrahydro-2-furoic acid), both of which are accessible from the hemicellulosic fraction of biomass. Conversion to the corresponding acid chlorides using thionyl chloride, followed by esterification with dipropylene glycol, afforded the target diesters in 72% and 86% yields. Both compounds were obtained in high purities (≥99.0% by ^1^H NMR; ≥98.0% by GC-MS).

### 3.2. Thermal Properties of (Bio)-Plasticizers

The melting temperatures of the developed bio-plasticizers were determined via DSC, and the results are presented in Table 1. The melting temperature indicates whether the plasticizer will be solid or liquid at room temperature, which affects the handling and processing conditions via melt compounding. Ideally, the plasticizers should be molten or sufficiently mobile to mix homogeneously with the polymer matrix and obtain effective plasticization. Commercial plasticizers typically possess very low melting temperatures (usually below −30 °C) and are liquid at room temperature [29]. The melting temperatures of the newly synthesized bio-plasticizers ranged from −64 to 62 °C. Although some bio-plasticizers displayed higher melting temperatures than conventional plasticizers, all remained well below the PLA processing temperature (190 °C), ensuring that they were molten during melt compounding. The bio-plasticizers Bis-F and Bis-THF, as well as MF/MA-C16, exhibited the lowest and highest melting temperatures. The presence of furanic aromatic rings in the Bis-F bio-plasticizer resulted in a melting temperature (T_m_ = −37 °C) comparable to that of the fossil-based aromatic counterpart, DPGDB (T_m_ = −40 °C) [30]. Aromatic rings promote stronger intermolecular interactions and greater molecular rigidity, which generally increases the melting temperature. In contrast, hydrogenation of the aromatic rings in the Bis-THF bio-derivative increases molecular flexibility, leading to a lower melting temperature (T_m_ = −64 °C). Furthermore, the inclusion of a methyl group in the bicyclic oxa-bridged ring resulted in a decrease in the T_m_ values, while the presence of a second methyl group led to a further reduction. This behavior was previously described by C. Plass et al. [18] when comparing a methyl-substituted bicyclic oxa-bridged ring bearing 2-ethylhexyl diester alkyl chains with its non-substituted plasticizer variant. In contrast, the increase in the alkyl chains length in the diesters resulted in higher T_m_ values of the investigated bio-plasticizers. Howell and Lazar reported a similar trend for their bio-based plasticizer esters derived from 2,5-bis-(hydroxymethyl)furan [31].

Thermogravimetric analysis was performed to evaluate the thermal stability of the newly developed bio-plasticizers. This aspect is also particularly important for plasticizers incorporated into polymers via melt compounding, as they must remain stable at polymer processing temperatures. Thermal instability can lead to decomposition or volatilization of the plasticizer, which can cause gas formation and reduced plasticization efficiency [32]. In this work, the (bio)-plasticizers were incorporated in PLA by melt compounding at 190 °C; therefore, they must exhibit thermal stability above this temperature.

**Figure 3:**
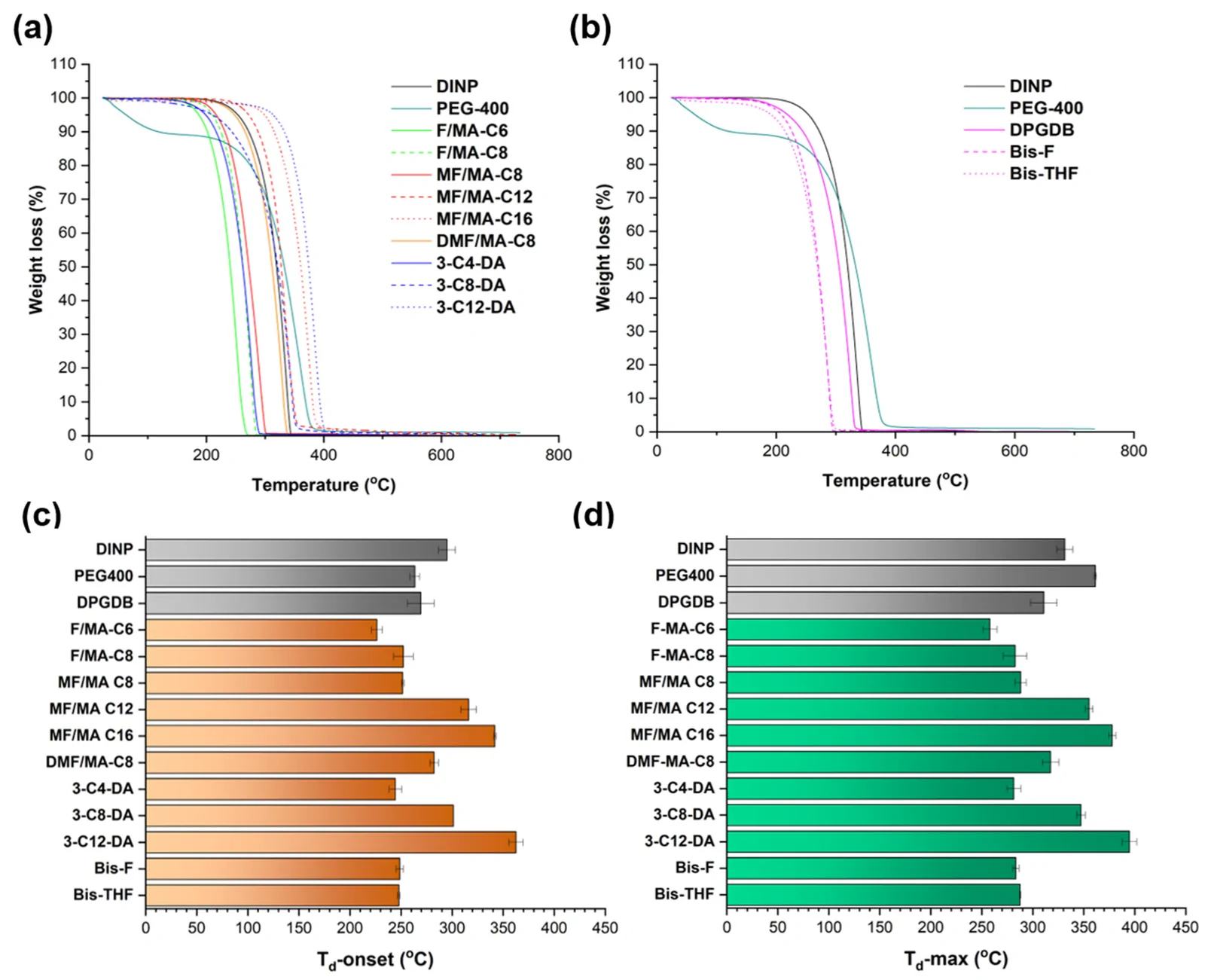
Thermal degradation properties: (**a**) TGA thermograms of bicyclic diester bio-plasticizers in comparison with DINP and PEG-400; (**b**) TGA thermograms of bio-based di(propylene glycol) dibenzoate derivatives in comparison with DINP, PEG-400, and DPGDB; (**c**) T_d_-onset values; and (**d**) T_d_-max values.

Thermograms of the developed bicyclic diester bio-plasticizers and the DPGDB bio-plasticizer derivatives are shown in Figure 3a and Figure 3b, respectively, while the T_d_-onset and T_d_-max values are shown in Figure 3c and Figure 3d, respectively. Conventional plasticizers DINP, PEG-400, and DPGDB were also included in Figure 3 for comparison purposes. All the studied (bio)-plasticizers showed a single-step weight-loss pattern in the thermograms except for PEG-400, which exhibited a two-step weight-loss pattern [31]. This response may be attributed to the evaporation/volatilization of lower molecular weight oligomers and moisture in the first weight-loss step [33].

As shown in Figure 3c, the degradation onset temperatures of all the investigated (bio)-plasticizers were above 190 °C, the selected PLA processing temperature, indicating that they maintain sufficient thermal stability under the processing conditions. The T_d_-onset temperatures of the developed bio-plasticizers varied between 226 and 363 °C, while the conventional plasticizers (DINP, PEG-400, and DPGDB) also exhibited values within this range, evidencing their suitability as potential substitutes for fossil-based plasticizers.

Moreover, for the bio-based plasticizers containing the MF/MA structure, increasing the length of alkyl chains in the diester groups resulted in an increase in the T_d_-onset of approximately 90 °C between the shortest (C8) and longest (C16) alkyl chains. A similar trend was exhibited for the bio-plasticizers featuring the F/MA structure, where the T_d_-onset values increased when moving from C6 to C8 alkyl chains. The same tendency was also found with the bio-plasticizers 3-C4-DA (T_d_-onset ~245 °C) and 3-C12-DA (T_d_-onset ~363 °C). This observed increase in thermal stability of the bio-plasticizers with longer alkyl chain lengths was consistent with trends reported elsewhere [31,34,35]. Among the di(propylene glycol) plasticizer derivatives, the bio-based Bis-THF and its hydrogenated counterpart, Bis-F, displayed comparable thermal stability with T_d_-onsets at approximately 248 °C, as well as the conventional DPGDB plasticizer, with slightly higher values at approximately 269 °C.

Additionally, the maximum thermal degradation temperatures of the investigated (bio)-plasticizers (Figure 3d) followed a similar trend to the T_d_-onset values and were approximately 30–47 °C higher. An exception was observed for PEG-400, which exhibited a difference of about 100 °C. The differences between the T_d_-onset and T_d_-max values for the investigated (bio)-plasticizers are presented in Table S1 (Supplementary Materials).

The developed bio-based plasticizers were evaluated in PLA, for which the T_d_-onset was determined to be 348.3 ± 9.1 °C. The comparatively lower thermal stability of PLA, relative to many conventional thermoplastics, is primarily attributed to the presence of hydrolysable ester linkages in its backbone, which are susceptible to thermal degradation through chain scission and transesterification reactions [36]. Nevertheless, the onset degradation temperature remains well above the processing temperature used in this study (190 °C), confirming that PLA is suitable for melt processing and for the incorporation of the developed bio-based plasticizers.

### 3.3. Theoretical Compatibility Between Plasticizers and Polymers via Hansen Solubility Parameters

Hansen Solubility Parameters (HSPs) are frequently used to predict the compatibility of additives with polymers [23]. In this work, HSPs were employed to theoretically evaluate the compatibility of the newly developed bio-based plasticizers with two frequently plasticized polymers, PLA and PVC. Although HSPs provide a useful first approximation of compatibility, plasticization efficiency also depends on factors such as molecular flexibility, diffusion behavior, free volume effects, and specific intermolecular interactions.

The HSP approach is based on the “like dissolves like” principle, where substances with similar HSPs have a high affinity for each other. The basis of HSPs is that the total cohesion energy of a substance consists of three interaction parameters, expressed in MPa^1/2^: dispersion (δ_d_), polar (δ_p_), and hydrogen bonding (δ_h_). Each substance (polymer or additive) can be represented as a point in a 3D Hansen space. The distance between two points, in this case plasticizer and polymer, can be calculated by the HSP distance (*R*_a_) as follows:

(1) $${\left(R_{a}\right)}^{2}=4{\left(\delta_{d,2}-\delta_{d,1}\right)}^{2}+{\left(\delta_{p,2}-\delta_{p,1}\right)}^{2}+{\left(\delta_{h,2}-\delta_{h,1}\right)}^{2}$$

where subscripts 1 and 2 represent substances 1 and 2, respectively. Additionally, based on the HSP sphere of the polymer with radius *R*_0_, the Relative Energy Difference (*RED*) can be defined as:

(2) RED=RaR0

When *RED* ≤ 1, the plasticizer is located inside the HSP sphere of the polymer, indicating a high affinity with the polymer. Otherwise, when the *RED* > 1, this indicates poor compatibility between the polymer and plasticizer.

**Figure 4:**
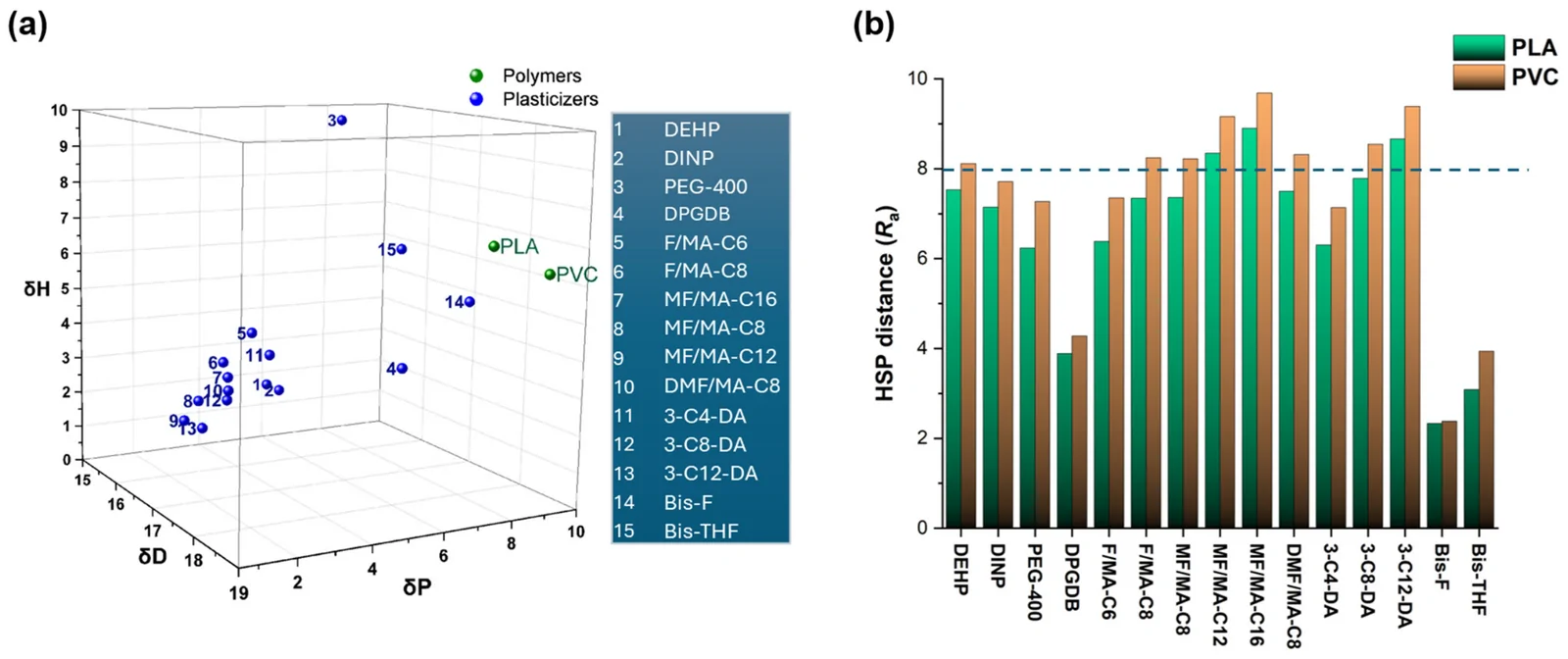
Theoretical compatibility between (bio)-plasticizers and polymers (PLA and PVC): (**a**) 3D Hansen space with the three contributions δD, δP, and δH of (bio)-plasticizers and polymers (PLA and PVC); and (**b**) HSP distance (*R*_a_) between the (bio)-plasticizers and polymers PLA and PVC. The dashed line indicates *R*_a_ = 8; therefore, good compatibility with the PLA/(bio)-plasticizer or PVC/(bio)-plasticizer is expected if *R*_a_ < 8.

The newly developed bio-plasticizers, as well as the conventional plasticizers such DEHP, DINP, PEG-400, and DPGDB, were included in the study. The HSPs of the (bio)-plasticizers were calculated using the group contribution method (Y-MB) of the HSPiP software (5th edition). The locations of the (bio)-plasticizers and polymers (PLA and PVC) in the 3D Hansen space are depicted in Figure 4a, and the distances between the (bio)-plasticizers and PLA and PVC are shown in Figure 4b. The calculated HSPs of the (bio)-plasticizers are depicted in Table S2 in the Supplementary Materials. According to the HSPiP software, the solubility sphere radius (*R*_o_) is eight for both PLA and PVC; hence, (bio)-plasticizers could be considered compatible with these polymers when the *R*_a_ values are less than eight (*RED* < 1).

As illustrated in Figure 4b, the HSP distance of the (bio)plasticizers from PVC was greater than that from PLA, suggesting that these investigated (bio)-plasticizers may exhibit higher compatibility with PLA than with PVC. The fossil-based DPGDB plasticizer and its bio-derivatives (Bis-F and Bis-THF) were predicted to exhibit the shortest HSP distances toward both PLA and PVC. Among these, the Bis-F bio-plasticizer presented the shortest distance to the two polymers, with *R*_a_ values of 2.3 for PLA and 2.4 for PVC, suggesting the highest predicted compatibility with these polymers. This can be attributed to the greater polarity of DPGDB and its synthesized bio-derivatives compared to the other plasticizers evaluated. Furthermore, these new bio-plasticizer alternatives (Bis-F and Bis-THF) showed higher affinity toward PLA and PVC than their fossil-based counterpart (DPGDB); hence, they could be considered as attractive substitutes. The bicyclic diester bio-plasticizers exhibit a dual structure, combining polar ester groups with non-polar alkyl chains. The results indicated that an increase in the alkyl chains length of the bio-plasticizers from six (F/MA-C6) to eight carbons (F/MA-C8), from eight (MF/MA-C8) to sixteen carbons (MF/MA-C16), and from four (3-C4-DA) to twelve carbons (3-C12-DA) leads to an increase in the distance between bio-plasticizer and the studied polymers; therefore, a decrease in the predicted affinity of these bio-plasticizers with the polymers is expected. In addition, the bio-plasticizers containing longer alkyl chains (C12 and C16) presented HSP distances to PLA and PVC higher than eight, suggesting limited compatibility as they lie outside the respective polymer solubility spheres. An increase in alkyl chain length enhances the nonpolar character of the bio-plasticizers, thereby reducing their compatibility with PVC and PLA, which exhibit moderate polarity [37]. The incorporation of a third ether alkyl chain (C8) in the bicyclic oxa-bridged ring (F/MA-C8 vs. 3-C8-DA) also led to a decrease in the expected compatibility between the bio-plasticizer and polymer. The inclusion of a single methyl group in the bicyclic oxa-bridged ring (F/MA-C8 vs. MF/MA-C8) caused only minor variations in the bio-plasticizer–polymer HSP distance, while the addition of two methyl groups (DMF/MA-C8) led to a slight increase in the HSP distance, implying diminished compatibility of the bio-plasticizer with the polymer matrix. The bicyclic diester bio-plasticizers with C8 alkyl chains exhibited HSP distances toward PLA and PVC comparable to those of phthalate plasticizers (DEHP and DINP), whereas PEG-400 presented lower values. These findings indicate that these bio-based variants have comparable compatibility with PLA and PVC to that of the evaluated phthalate plasticizers and thus could serve as promising alternatives.

### 3.4. Thermomechanical Properties of PLA–(Bio)-Plasticizer Blends

The effectiveness of the newly synthesized bio-plasticizers was evaluated in PLA due to their biodegradability, renewable nature, and relevance in sustainable polymer applications. Moreover, the HSP’ss indicated greater compatibility of the developed bio-plasticizers with PLA than with PVC. Although PVC remains one of the most widely plasticized commercial polymers, the increasing focus on sustainable and bio-based materials makes PLA a more appropriate platform for evaluating the performance of these newly synthesized bio-plasticizers. To the best of our knowledge, the developed furfural-derived compounds have not previously been reported as plasticizers, nor have they been systematically evaluated in PLA. The produced bio-plasticizers were incorporated in PLA via melt compounding at a load of 15 wt%. This value was selected as a representative concentration within the commonly reported PLA plasticization range (10–30 wt%), enabling a direct comparison between all candidates while limiting the scope of experimental variables [15]. The selected concentration therefore allowed a systematic evaluation of all eleven plasticizers under identical conditions, facilitating the identification of the most promising candidates. The conventional plasticizers DINP, PEG-400, and DPGDB were selected as reference plasticizers due to their widespread industrial use, and were also tested experimentally for comparison with the newly synthesized bio-plasticizers.

**Figure 5:**
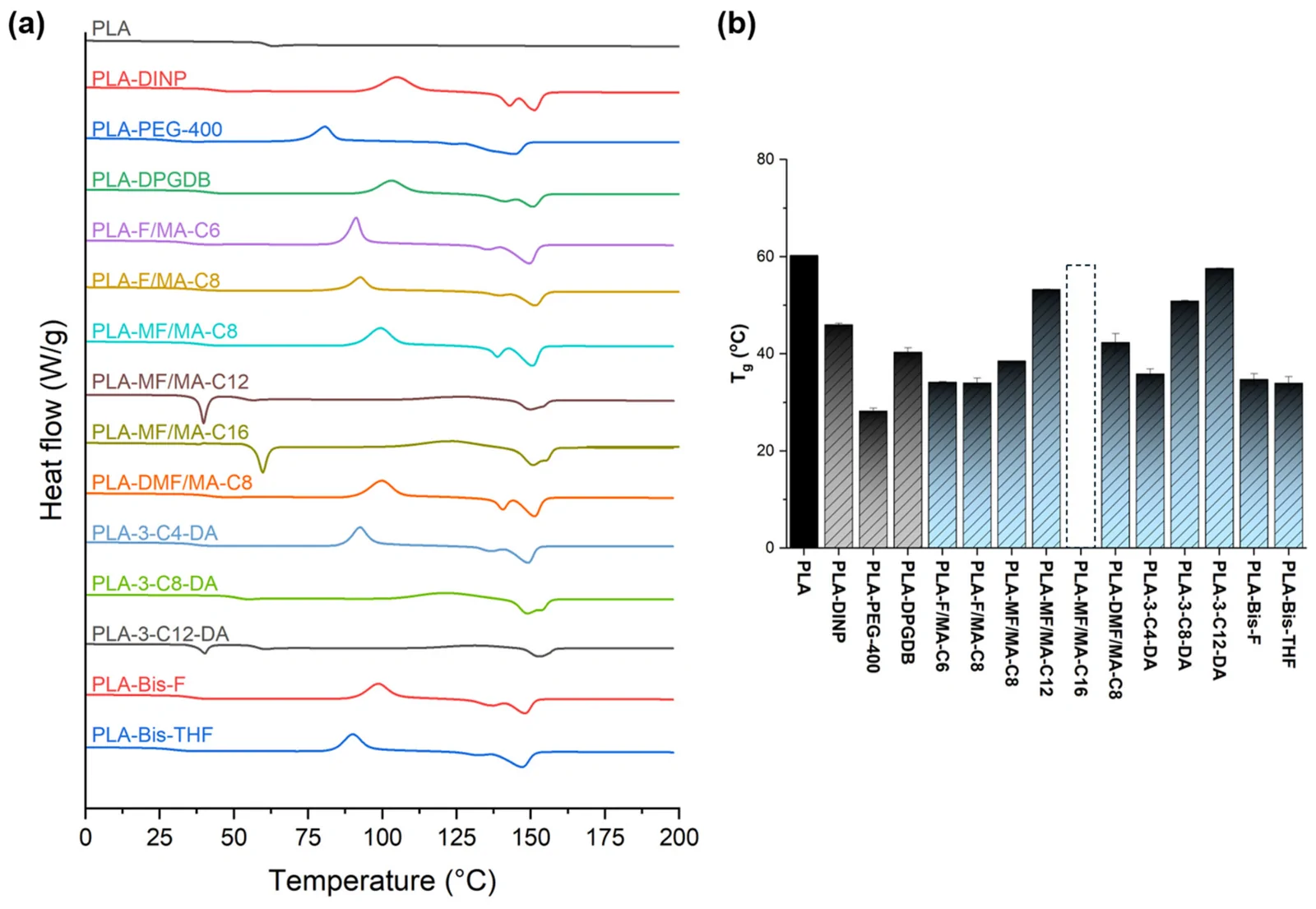
Differential scanning calorimetry (DSC): (**a**) curves (exo up) of second heating run, and (**b**) glass transition temperature (T_g_) for neat PLA and the PLA–(bio)-plasticizer blends at 15 wt% of (bio)-plasticizer loading. Dashed line indicates the potential T_g_ value for the PLA-MF/MA-C16 blend.

The DSC curves of the second heating run of neat PLA and its blends with the (bio)-plasticizers are presented in Figure 5a, and the resulting glass transition temperatures (T_g_) are depicted in Figure 5b. Plasticizers decrease the glass transition temperature (T_g_) of polymers by weakening intermolecular interactions and increasing the free volume between polymer chains, which enhances segmental chain mobility. As a result, the polymers transition from a glassy to a rubbery state at lower temperatures. Therefore, a reduction in T_g_ indicates the plasticizing effect of the incorporated (bio)-plasticizer within the PLA matrix. The glass transition temperature of neat PLA was found to be approximately 60.2 °C, which was consistent with the value provided by the supplier [38]. Unplasticized PLA did not exhibit detectable cold crystallization (T_cc_) or melting (T_m_) peaks during the second heating run, indicating that the polymer remained essentially amorphous due to its inherently slow crystallization kinetics and limited chain mobility. Among the different PLA–(bio)-plasticizer blends, PLA-PEG-400, PLA-F/MA-C6, PLA-F/MA-C8, PLA-Bis-F, and PLA-Bis-THF displayed the most pronounced reductions in glass transition temperatures, reaching values between 28 and 35 °C, compared to neat PLA. These decreases in T_g_ values were also reported by Benkraled et al. [21] in different PLA-PEG-400 blends, where a T_g_ of 27 °C was obtained with 15 wt% loading of PEG-400, which was comparable to the values observed in our system. This reduction in T_g_ values indicates enhanced polymer chain mobility due to plasticization and suggests good compatibility between these plasticizers and PLA.

The PLA blends containing the conventional DPGDB plasticizer presented higher T_g_ values than those with its bio-based derivatives (Bis-F and Bis-THF), highlighting the potential of these novel bio-based plasticizers. Significant reductions in T_g_ (36–42 °C) were also observed for PLA-DPGDB, PLA-DMF/MA-C8, PLA-MF/MA-C8, and PLA-3-C4-DA blends. These PLA–(bio)-plasticizer blends, which showed reduced T_g_ values compared to neat PLA, were also predicted to exhibit a higher affinity for PLA based on Hansen Solubility Parameters. Moreover, upon the addition of these promising (bio)-plasticizers to PLA, both T_cc_ (ranging from 80 to 105 °C) and T_m_ (ranging from 143 to 151 °C) peaks became evident, as illustrated in Figure 5a. This phenomenon could be explained by the increased polymer chain flexibility and mobility, which facilitates molecular rearrangement into ordered crystalline structures, suggesting that these (bio)-plasticizers promote the crystallization of PLA [21,39]. The incorporation of one or two methyl groups in the oxa-bicyclic ring of the di-ester bio-plasticizers increased the T_g_ of the PLA blends, suggesting enhanced plasticization efficiency of the non-substituted F/MA-C8 bio-plasticizer. In addition, increasing the di-ester alkyl chain length of the bio-plasticizers resulted in higher T_g_ values than with shorter alkyl chains, indicating lower plasticization efficiency [34]. This response could be attributed to the reduced compatibility between these bio-plasticizers and PLA, likely resulting from the increased hydrophobicity imparted by the alkyl chains, which was also consistent with their lower predicted affinity based on HSP analysis. This lower compatibility of the bio-plasticizers with longer alkyl chains (C12, C16) with PLA was also evident from the DSC curves (Figure 5a) for PLA-MF/MA-C12, PLA-MF/MA-C16, and PLA-3-C12-DA, as they exhibited an endothermic melting peak corresponding to the melting temperatures of the individual bio-plasticizers with no crystallization peaks, suggesting that the PLA remained amorphous. These results highlight that only plasticizers capable of sufficiently increasing PLA chain mobility can induce crystallization in the polymer. The melting peak of the MF/MA-C16 bio-plasticizer overlapped with the expected T_g_ region of the PLA-MF/MA-C16 blend, making the determination of the glass transition temperature for this blend not feasible.

**Figure 6:**
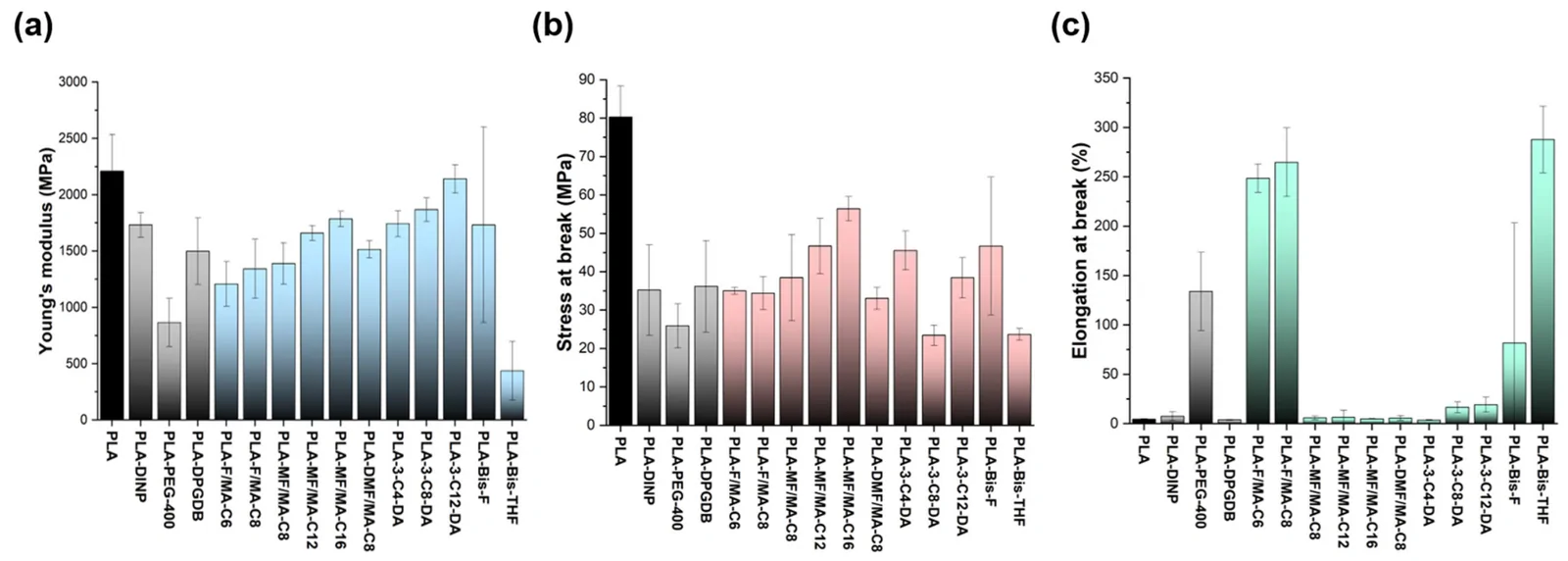
Mechanical properties of neat PLA and PLA–(bio)-plasticizer blends at 15 wt% of (bio)-plasticizer loading: (**a**) Young’s modulus, (**b**) stress at break, and (**c**) elongation at break.

The mechanical properties of unplasticized PLA and its blends with the investigated (bio)-plasticizers were evaluated by tensile testing. The Young’s modulus, stress at break, and elongation at break values were obtained and are depicted in Figure 6a, Figure 6b and Figure 6c, respectively. These properties were evaluated to determine whether the reduction in T_g_ also translates into improved flexibility and ductility of PLA. Effective plasticizers generally reduce Young’s modulus and the stress at break while increasing the elongation at break, indicating that the polymer matrix has become softer and more flexible [8]. Young’s modulus, which reflects the stiffness of a material, was approximately 2200 MPa for neat PLA [40], whereas most of the investigated PLA–(bio)-plasticizer blends exhibited lower values than PLA, reflecting reduced stiffness. The greatest reductions in Young’s modulus values were observed for PLA-Bis-THF (438 MPa) and PLA-PEG-400 (867 MPa), corresponding to relative decreases compared to PLA of approximately 80% and 61%, respectively, indicating increased chain mobility and plasticization of PLA. Comparable reductions in Young’s modulus were also reported by Benkraled et al. [21] in different PLA-PEG-400 blends. A notable reduction in Young’s modulus between 32% and 45% compared to neat PLA was also observed for the PLA-F/MA-C6, PLA-F/MA-C8, PLA-MF/MA-C8, and PLA-DMF-MA/C8 blends.

The stress at break (Figure 6b) reflects the maximum tensile stress a material can withstand before failure, and it is inversely influenced by the presence of effective plasticizers. A balanced plasticizer system maintains sufficient stress at break while improving flexibility. As expected, neat PLA exhibited the highest stress at break (80 MPa), in agreement with previously reported values [41], reflecting its rigid structure and limited chain mobility. PLA blends containing PEG-400, 3-C8-DA, and Bis-THF bio-plasticizers depicted the lowest values of stress at break (23–26 MPa), whereas the PLA-MF/MA-C12 and PLA-MF/MA-C16 blends displayed the highest values (45–57 MPa), suggesting relatively lower chain mobility of the polymer matrix [42].

The elongation at break manifests the ductility of a material and serves as key indicator of plasticization efficiency. The results from Figure 6c show that unplasticized PLA exhibited an elongation at break of approximately 4.5%, whereas the presence of the F/MA-C6, F/MA-C8 and Bis-THF bio-plasticizers increased the elongation values remarkably to between 248% and 288%. These values exceeded those obtained with the conventional plasticizer PEG-400 (134%). This pronounced increase in the elongation at break indicates improved plasticizer efficiency, reflecting enhanced chain mobility and increased polymer flexibility. The PLA-Bis-F blend showed a large standard deviation, suggesting that sample inhomogeneity and microscopic defects led to large variability in mechanical performance. This variability may indicate local heterogeneity in plasticizer distribution, incipient phase separation, or processing-induced specimen variability. Furthermore, moderate increases in the elongation at break were observed for the PLA-3-C8-CA and PLA-3-C12-DA blends, reaching approximately 16% and 19%, respectively. Nevertheless, the incorporation of one or two methyl groups in the bicyclic oxa-bridged ring of the bio-plasticizers (F/MA-C8 vs. MF/MA-C8 and DMF/MA-C8) resulted in no improvement of the elongation at break values for these PLA–bio-plasticizer blends compared to neat PLA, evidencing limited plasticization efficiency. These findings highlight how a small structural change in the plasticizer structure drastically affects their efficiency. The methyl groups introduce steric hindrance around the bicyclic oxa-bridged ring, reducing the ability of the plasticizer to intercalate and separate polymer chains, which is essential for effective plasticization. Furthermore, as indicated by HSP analysis, the incorporation of methyl groups into the bicyclic oxa-bridged ring reduces the polarity of the plasticizer, decreasing its compatibility with PLA. Additionally, the added methyl groups increase the rigidity of the molecule, limiting its ability to adapt to the polymer’s free volume. An increase in the di-ester alkyl chain length of the bio-plasticizers from C8 to C16 (MF/MA-C8, MF/MA-C12, and MF/MA-C16) similarly did not result in enhanced plasticization efficiency of PLA, in concordance with their reduced predicted affinity with PLA via HSPs. Surprisingly, the incorporation of the conventional fossil-based plasticizers DINP and DPGDB into PLA resulted in elongation at break values comparable to those of neat PLA, indicating their limited effectiveness for PLA plasticization under the tested conditions. The relatively poor performance of DINP and DPGDB may be related to the presence of rigid benzyl moieties. Their reduced conformational flexibility may limit the ability of these plasticizer molecules to interact effectively with the polymer matrix, thereby diminishing their plasticization efficiency. Variable elongation at break values were reported for plasticized PLA in the literature, largely depending on the nature of the plasticizer (e.g., chemical structure, molecular weight, and polarity) and its concentration [15,43].

In conclusion, the reduced T_g_, Young’s modulus, and stress at break, together with the remarkable increase in elongation at break values compared to neat PLA, identify the novel F/MA-C6, F/MA-C8, and Bis-THF bio-plasticizers as the most promising candidates for PLA under the tested conditions.

**Figure 7:**
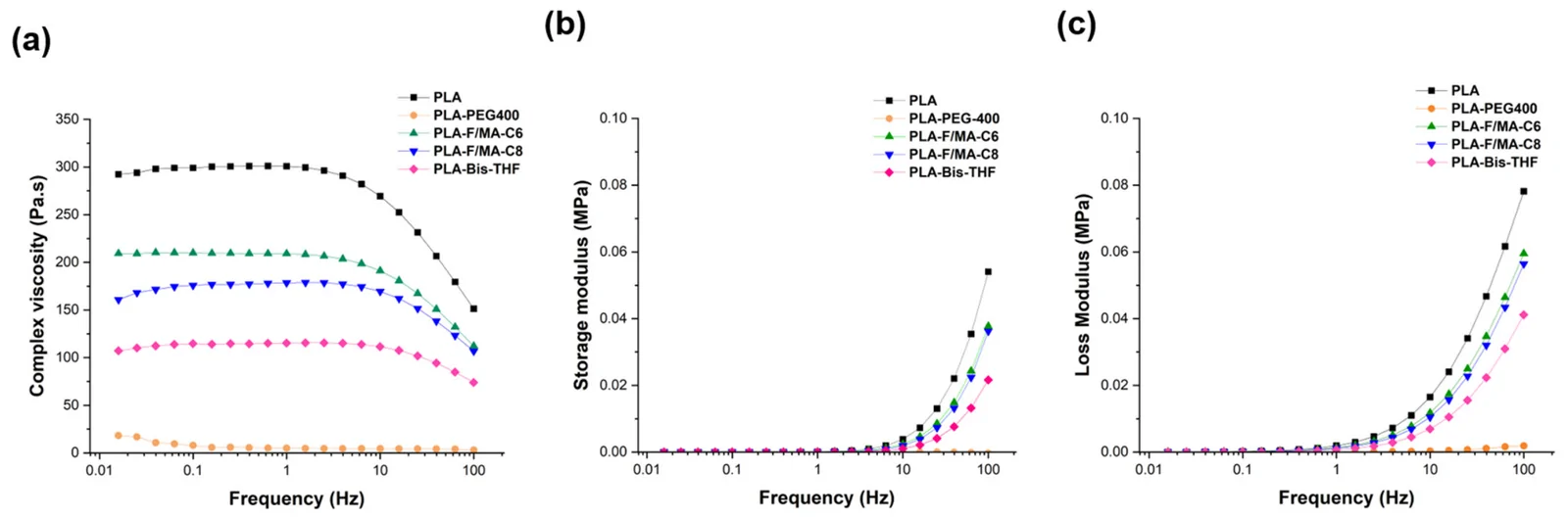
Frequency-dependent (**a**) complex viscosity, (**b**) storage modulus, and (**c**) loss modulus behaviors of PLA and the most promising investigated PLA–bio-plasticizers blends at 190 °C.

Complex viscosity, storage modulus, and loss modulus as a function of frequency were determined for neat PLA and the most promising investigated PLA–bio-plasticizers blends, and the results are illustrated in Figure 7a, Figure 7b and Figure 7c, respectively. These measurements provide insight into the frequency-dependent viscoelastic behavior of the PLA melts, reflecting chain mobility, energy dissipation, and processability [44]. Rheological measurements were performed at 190 °C, corresponding to the processing temperature during melt compounding and injection molding, thereby enabling the evaluation of melt viscoelastic behaviors of the most promising PLA/plasticizer formulations under representative processing conditions. The neat PLA exhibited a low-frequency complex viscosity plateau, followed by strong shear thinning at higher frequencies, indicating highly entangled viscoelastic properties and long relaxation times of the polymer [45]. The addition of the most promising synthesized bio-plasticizers and PEG-400 to PLA significantly reduced both the complex viscosity across the entire frequency range and the shear thinning at higher frequencies, indicating increased chain mobility and improved flowability. This behavior is a good indicator of increased plasticization [21]. The PLA-PEG-400 blend exhibited the lowest complex viscosity values, followed by the PLA-Bis-THF, PLA-F/MA-C8, and PLA-F/MA-C6 blends.

The storage modulus (G′) represents the elastic (solid-like) behavior, and measures how much energy the material can store during deformation, while the loss modulus (G″) represents the viscous (liquid-like) behavior and measures how much energy is dissipated as flow. As expected, unplasticized PLA presented the higher values of G′ and G″, which increased with higher frequencies, compared to the PLA–bio-plasticizer blends studied, as shown in Figure 7b and Figure 7c, respectively. The reduction in storage and loss moduli in the presence of the studied bio-plasticizers indicates that the material becomes less stiff and offers less resistance to flow, which is fundamental for predicting thermal processing behavior and guiding material design.

The most promising furan-based plasticizers combine a renewable origin, adequate thermal stability for melt processing, and superior plasticization efficiency compared to the reference plasticizers evaluated in this study. In addition, the systematic variation in molecular structure provides valuable insight into structure–property relationships, supporting the rational design of future bio-based plasticizers. The promising thermomechanical performance provides strong initial validation of the synthesized bio-based plasticizers. Nevertheless, further investigations, including migration or extraction tests, long-term aging, scanning electron microscopy (SEM), and temperature-sweep DMA, would be required to evaluate the long-term compatibility and retention of the plasticizers within the PLA matrix, as well as providing additional experimental validation of the HSP predictions. Further research should also investigate the biodegradation of the synthesized plasticizers and their influence on the biodegradation behavior of plasticized PLA to further assess the sustainability of the developed materials. Moreover, their potential for industrial implementation should be evaluated through toxicity screening and techno-economic and life cycle assessments, including assessments of the carbon footprint associated with the production, processing, and application of bio-plasticizers. Such an assessment would help determine their overall environmental benefits compared with conventional plasticizers and identify opportunities for further reductions in their carbon footprints.

The synthesis of these novel bio-based plasticizers also opens opportunities for their application in bio-based and biodegradable polymers beyond PLA. Furthermore, as renewable alternatives to conventional fossil-based plasticizers, they may also have potential for use in conventional polymer systems. Further studies would be required to evaluate their compatibility and plasticization efficiency in these polymer matrices.

## 4. Conclusions

In this paper, eleven novel bio-plasticizers, namely F/MA-C6, F/MA-C8, MF/MA-C8, MF/MA-C12, MF/MA-C16, DMF/MA-C8, 3-C4-DA, 3-C8-DA, 3-C12-DA, Bis-F, and Bis-THF, were synthesized from renewable resources, such as furfural derivatives, and their effects on the thermomechanical properties of PLA were investigated. These bio-plasticizers exhibit structural variability, enabling evaluation of the degree of substitution, the length of the alkyl chains of the bicyclic oxa-bridged diester candidates, and the substitution of fossil-based benzene rings with bio-based furan or its hydrogenated equivalent, tetrahydrofuran, in di(propylene glycol) derivatives. Comparisons with conventional plasticizers, such as DINP, PEG-400, and DPGDB, were also performed. These unique bio-plasticizers demonstrated sufficient thermal stability for melt compounding with the PLA. While HSPs do not provide a direct quantitative measurement of plasticization efficiency, it serves as a powerful tool for selecting the more compatible plasticizer additives with polymer. An increase in alkyl chain length and degree of substitution in the bicyclic oxa-bridged diester plasticizers led to reduced plasticization efficiency of PLA under the tested conditions, as evidenced by higher glass transition temperatures and lower elongation at break values. This effect could be attributed to diminished compatibility with PLA, consistent with the predictions by HSPs. Furthermore, the incorporation of furan or tetrahydrofuran moieties in place of benzene rings in the di(propylene glycol) derivatives resulted in improved compatibility with PLA, as evidenced by the HSPs, a reduced T_g_ and Young’s modulus, and an increased elongation at break, demonstrating improved plasticization of PLA. The PLA-F/MA-C6, PLA-F/MA-C8, and PLA-Bis-THF blends exhibited exceptional elongation at break, reaching values of 248%, 266%, and 289%, respectively, compared to 4.5% for unplasticized PLA under the tested conditions. Among the reference plasticizers, only PEG-400 exhibited effective plasticization under the tested conditions, reducing T_g_ to 28.2 °C and increasing elongation at break to 134%. These findings demonstrate that plasticization efficiency is strongly governed by the molecular structure of the plasticizer, as well as its compatibility and interactions with the PLA matrix. The promising PLA blends containing the synthesized bio-plasticizers also reduced both the complex viscosity and the shear thinning at higher frequencies compared to unplasticized PLA, demonstrating improved polymer chain mobility and flowability. Consequently, the candidates F/MA-C6, F/MA-C8, and Bis-THF emerged as the most promising bio-based plasticizers evaluated in this study, indicating their potential for application in flexible PLA formulations. Future work should focus on assessing migration behavior, long-term aging, toxicity, biodegradability, and techno-economic feasibility to ensure safety and long-term performance in practical applications.

## Figures and Tables

**Table 1:** Basic descriptions of developed bio-plasticizers.

Abbreviation	Full Name	Chemical Formula	Molecular Weight (g/mol)	Appearance ^1^	Melting Temperature (°C) ^2^
F/MA-C6	7-Oxabicyclo[2.2.1]heptane-2,3-dicarboxylic acid, 2,3-dihexyl ester	C_20_H_34_O_5_	354.5	Solid	34.5 ± 0.1
F/MA-C8	7-Oxabicyclo[2.2.1]heptane-2,3-dicarboxylic acid, 2,3-dioctyl ester	C_24_H_42_O_5_	410.6	Solid	38.5 ± 0.1
MF/MA-C8	7-Oxabicyclo[2.2.1]heptane-2,3-dicarboxylic acid, 1-methyl-, 2,3-dioctyl ester	C_25_H_44_O_5_	424.6	Solid	27.8 ± 0.1
MF/MA-C12	7-Oxabicyclo[2.2.1]heptane-2,3-dicarboxylic acid, 1-methyl-, 2,3-didodecyl ester	C_33_H_60_O_5_	536.8	Solid	40.8 ± 0.1
MF/MA-C16	7-Oxabicyclo[2.2.1]heptane-2,3-dicarboxylic acid, 1-methyl-, 2,3-dihexadecyl ester	C_41_H_76_O_5_	649.0	Solid	62.1 ± 0.2
DMF/MA-C8	7-Oxabicyclo[2.2.1]heptane-2,3-dicarboxylic acid, 1,4-dimethyl, 2,3-dioctyl ester	C_26_H_46_O_5_	438.6	Semi-solid	18.4 ± 0.3
3-C4-DA	Dibutyl 1-butoxymethyl-7-oxabicyclo[2.2.1] heptane-2,3- dicarboxylate	C_21_H_36_O_6_	384.5	Liquid	23.2 ± 0.9
3-C8-DA	Dioctyl 1-octoxymethyl-7-oxabicyclo[2.2.1] heptane-2,3- dicarboxylate	C_33_H_60_O_6_	552.8	Liquid	4.3 ± 0.1
3-C12-DA	Didodecyl 1-dodecyloxymethyl-7-oxabicyclo[2.2.1] heptane-2,3-dicarboxylate	C_45_H_84_O_6_	721.1	Solid	40.8 ± 0.1
Bis-F	1,1′-[oxybis(1-methyl-2,1-ethanediyl)] bisfuroate	C_16_H_18_O_7_	322.1	Liquid	−36.8 ± 0.2
Bis-THF	1,1′-[oxybis(1-methyl-2,1-ethanediyl)] bistetrahydrofuroate	C_16_H_26_O_7_	330.4	Liquid	−63.8 ± 0.6

^1^ At room temperature. ^2^ Determined by differential scanning calorimetry in the second heating run.

**Table 2:** Reaction yields and purities of the synthesized bio-plasticizers and their reaction intermediates.

Compound	Name	R_1_	R_2_	R_3_	R_4_	Yield (%)	Purity (%) ^1^H NMR	Purity (%) GC-MS
**3a**	7-oxabicyclo[2.2.1]hept-5-ene-2,3-dicarboxylic anhydride	H	H	-	-	73	-	-
**3b**	1-methyl-7-oxabicyclo[2.2.1]hept-5-ene-2,3-dicarboxylic anhydride	CH_3_	H	-	-	86	-	-
**3c**	1,4-dimethyl-7-oxabicyclo[2.2.1]hept-5-ene-2,3-dicarboxylic anhydride	CH_3_	CH_3_	-	-	70	-	-
**4a**	7-oxabicyclo[2.2.1]heptane-2,3-dicarboxylic anhydride	H	H	-	-	72	99.6	-
**4b**	1-methyl-7-oxabicyclo[2.2.1]heptane-2,3-dicarboxylic anhydride	CH_3_	H	-	-	87	99.9	-
**4c**	1,4-dimethyl-7-oxabicyclo[2.2.1]heptane-2,3-dicarboxylic anhydride	CH_3_	CH_3_	-	-	87	100.0	-
**6**	F/MA-C6	H	H	C_5_H_11_	-	62	≥98.0	≥99.0
**7**	F/MA-C8	H	H	C_7_H_15_	-	68	≥98.0	≥98.0
**8**	MF/MA-C8	CH_3_	H	C_7_H_15_	-	74	≥99.0	≥99
**9**	MF/MA-C12	CH_3_	H	C_11_H_23_	-	42	≥97.0	≥96.0
**10**	MF/MA-C16	CH_3_	H	C_15_H_31_	-	59	≥98.0	-
**11**	DMF/MA-C8	CH_3_	CH_3_	C_7_H_15_	-	86	≥98.0	-
**14a**	butyl-furfuryl ether	-	-	-	C_3_H_7_	81	-	-
**14b**	octyl-furfuryl ether	-	-	-	C_7_H_15_	106	-	-
**14c**	dodecyl-furfuryl ether	-	-	-	C_11_H_23_	89	-	-
**15a**	4-(butoxymethyl)-3a,4,7,7a-tetrahydro-4,7-epoxyisobenzofuran-1,3-dione	-	H	-	C_3_H_7_	82	-	-
**15b**	4-(octoxymethyl)-3a,4,7,7a-tetrahydro-4,7-epoxyisobenzofuran-1,3-dione	-	H	-	C_7_H_15_	53	-	-
**15c**	4-(dodecoxymethyl)-3a,4,7,7a-tetrahydro-4,7-epoxyisobenzofuran-1,3-dione	-	H	-	C_11_H_23_	72	-	-
**16a**	4-(butoxymethyl)hexahydro-4,7-epoxyisobenzofuran-1,3-dione	-	H	-	C_3_H_7_	79	-	-
**16b**	4-(octoxymethyl)hexahydro-4,7-epoxyisobenzofuran-1,3-dione	-	H	-	C_7_H_15_	64	-	-
**16c**	4-(dodecoxymethyl)hexahydro-4,7-epoxyisobenzofuran-1,3-dione	-	H	-	C_11_H_23_	53	-	-
**18**	3-C4-DA	-	H	C_3_H_7_	C_3_H_7_	77	≥98.0	≥98.0
**19**	3-C8-DA	-	H	C_7_H_15_	C_7_H_15_	71	≥95.0	-
**20**	3-C12-DA	-	H	C_11_H_23_	C_11_H_23_	62	≥95.0	-
**23**	Bis-F	-	-	-	-	72	≥99.0	≥98.0
**24**	Bis-THF	-	-	-	-	86	≥99.0	≥98.0

## Data Availability

The original contributions presented in the study are included in the article. Further inquiries can be directed at the corresponding author.

## Supplementary Materials

The following supporting information can be downloaded at: https://www.mdpi.com/article/10.3390/polym18172070/s1, Figure S1. ^1^H NMR spectra of F/MA-C6 bio-plasticizer (with dimethyl fumarate as internal standard); Figure S2. ^1^H NMR spectra of F/MA-C8 bio-plasticizer (with dimethyl fumarate as internal standard); Figure S3. ^1^H NMR spectra of MF/MA-C8 bio-plasticizer (with dimethyl fumarate as internal standard); Figure S4. ^1^H NMR spectra of MF/MA-C12 bio-plasticizer (with dimethyl fumarate as internal standard); Figure S5. ^1^H NMR spectra of MF/MA-C16 bio-plasticizer (with dimethyl fumarate as internal standard); Figure S6. ^1^H NMR spectra of DMF/MA-C8 bio-plasticizer (with dimethyl fumarate as internal standard); Figure S7. ^1^H NMR spectra of 3-C4-DA bio-plasticizer (with dimethyl fumarate as internal standard); Figure S8. ^1^H NMR spectra of 3-C8-DA bio-plasticizer (with nitromethane as internal standard); Figure S9. ^1^H NMR spectra of 3-C12-DA bio-plasticizer (with nitromethane as internal standard); Figure S10. ^1^H NMR spectra of Bis-F bio-plasticizer (with dimethyl fumarate as internal standard); Figure S11. ^1^H NMR spectra of Bis-THF bio-plasticizer (with p-xylene as internal standard); Figure S12. MS spectra of F/MA-C6 bio-plasticizer (*m*/*z* 33-400); Figure S13. MS spectra of F/MA-C8 bio-plasticizer (*m*/*z* 33-400); Figure S14. MS spectra of MF/MA-C8 bio-plasticizer (*m*/*z* 33-600); Figure S15. MS spectra of MF/MA-C12 bio-plasticizer (*m*/*z* 33-600); Figure S16. MS spectra of 3-C4-DA bio-plasticizer (*m*/*z* 33-400).; Figure S17. MS spectra of Bis-F bio-plasticizer (*m*/*z* 33-600); Figure S18. MS spectra of Bis-THF bio-plasticizer (*m*/*z* 33-600); Table S1. Comparison of thermal degradation parameters (T_d_-onset vs. T_d_-max); Table S2. Hansen Solubility Parameters of (bio)-plasticizers and polymers (PVC and PLA).

## Abbreviations

The following abbreviations are used in this manuscript:

PLA	Polylactic acid
PVC	Polyvinyl chloride
DEHP	Di(2-ethylhexyl) phthalate
DINP	Diisononyl phthalate
PEG-400	Polyethylene glycol 400
MF/MA-C8	7-Oxabicyclo[2.2.1]heptane-2,3-dicarboxylic acid, 1-methyl-, 2,3-dioctyl ester
MF/MA-C12	7-Oxabicyclo[2.2.1]heptane-2,3-dicarboxylic acid, 1-methyl-, 2,3-didodecyl ester
MF/MA-C16	7-Oxabicyclo[2.2.1]heptane-2,3-dicarboxylic acid, 1-methyl-, 2,3-dihexadecyl ester
F/MA-C6	7-Oxabicyclo[2.2.1]heptane-2,3-dicarboxylic acid, 2,3-dihexyl ester
F/MA-C8	7-Oxabicyclo[2.2.1]heptane-2,3-dicarboxylic acid, 2,3-dioctyl ester
DMF/MA-C8	7-Oxabicyclo[2.2.1]heptane-2,3-dicarboxylic acid, 1,4-dimethyl, 2,3-dioctyl ester
3-C4-DA	Dibutyl 1-butoxymethyl-7-oxabicyclo[2.2.1]heptane-2,3-dicarboxylate
3-C8-DA	Dioctyl 1-octoxymethyl-7-oxabicyclo[2.2.1]heptane-2,3-dicarboxylate
3-C12-DA	Didodecyl 1-dodecyloxymethyl-7-oxabicyclo[2.2.1]heptane-2,3-dicarboxylate
Bis-F	1,1′-[oxybis(1-methyl-2,1-ethanediyl)] disfuroate
Bis-THF	1,1′-[oxybis(1-methyl-2,1-ethanediyl)] distetrahydrofuroate
DPGDB	Dipropylene glycol dibenzoate
HSP	Hansen solubility parameter
TGA	Thermogravimetric analysis
DSC	Differential scanning calorimetry
T_g_	Glass transition temperature
T_m_	Melting temperature
T_cc_	Cold crystallization temperature
T_d_-onset	Degradation temperature onset
T_d_-max	Maximum degradation temperature
