# Supplementary material for: Synthesis and Evaluation of Eleven Novel Renewable Plasticizers for Polylactic Acid (PLA): Linking Molecular Structure to Performance
Source: Polymers (Basel). 2026 Aug 26;18(17):2070. doi: 10.3390/polym18172070 (PMC13568462; doi:10.3390/polym18172070)
Supplement: Supplementary file 1 [file polymers-18-02070-s001.zip › polymers-4480633-supplementary.pdf]

Supplementary information

Synthesis and Evaluation of Eleven Novel Renewable Plasticizers for Polylactic Acid (PLA): Linking Molecular Structure to Performance

Ferry Oomen [1], Marc Crockatt [2], Eric Mattheussens [1], Ivan Bakker [2], Michał Pstrowski [2], Maddalena Logrieco [1], Moctar Coulibaly [1], Han van Kasteren [1,*], Sandra Corderí Gándara [1,3,*]

[1] Centre of Expertise Materials and Energy Transition (MNEXT), Lovensdijkstraat 63, 4818 AJ Breda, The Netherlands
[2] Department of Sustainable Process and Energy Systems, TNO, Kessler Park 1, 2288 GH Rijswijk, The Netherlands
[3] Flemish Institute for Technological Research (VITO N.V.), Materials and Chemistry Unit (MatCh), Boeretang 200, 2400 Mol, Belgium
* Correspondence: jmn.vankasteren@avans.nl (H.v.K.); s.corderigandara@avans.nl (S.C.G.)

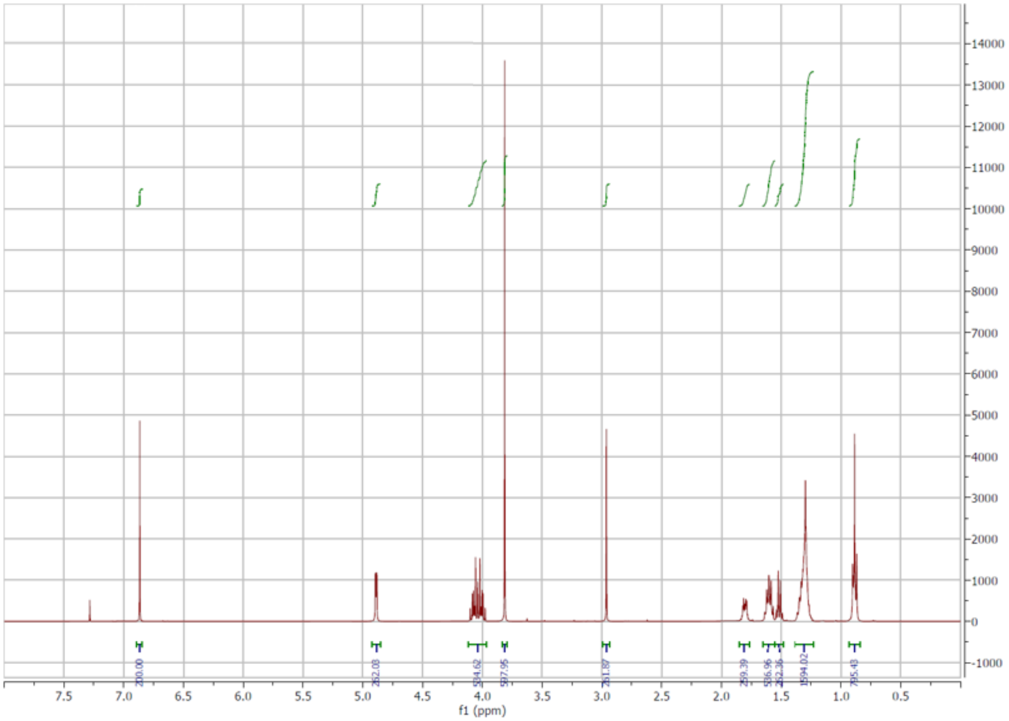

Figure S1. ^{1}H NMR spectra of F/MA-C6 bio-plasticizer (with dimethyl fumarate as internal standard).

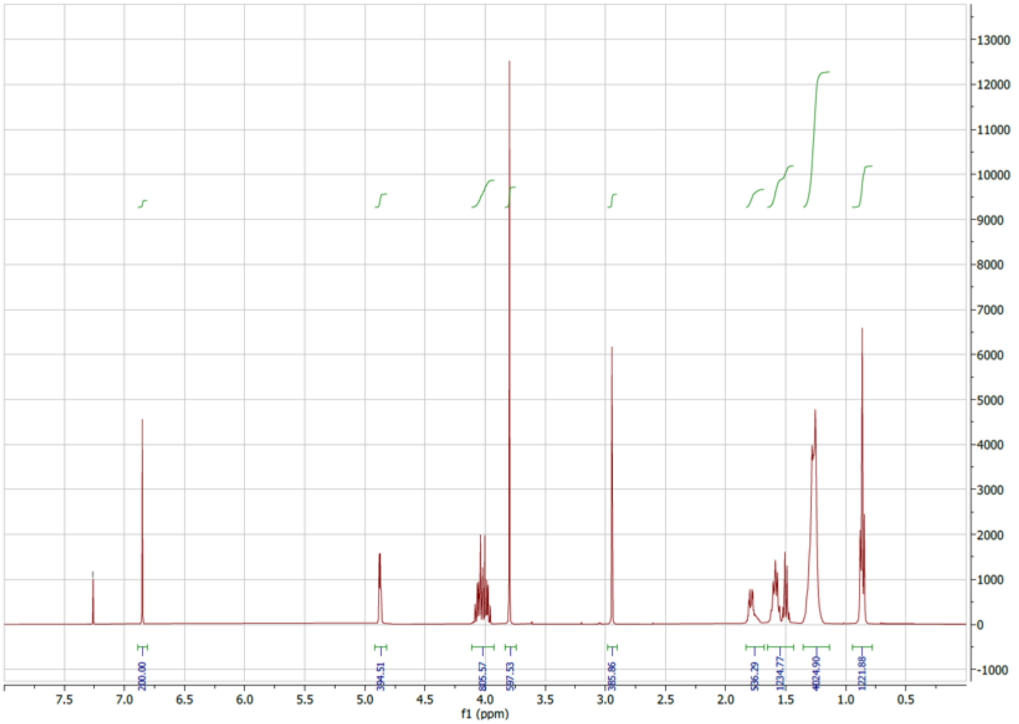

Figure S2. ^{1}H NMR spectra of F/MA-C8 bio-plasticizer (with dimethyl fumarate as internal standard).

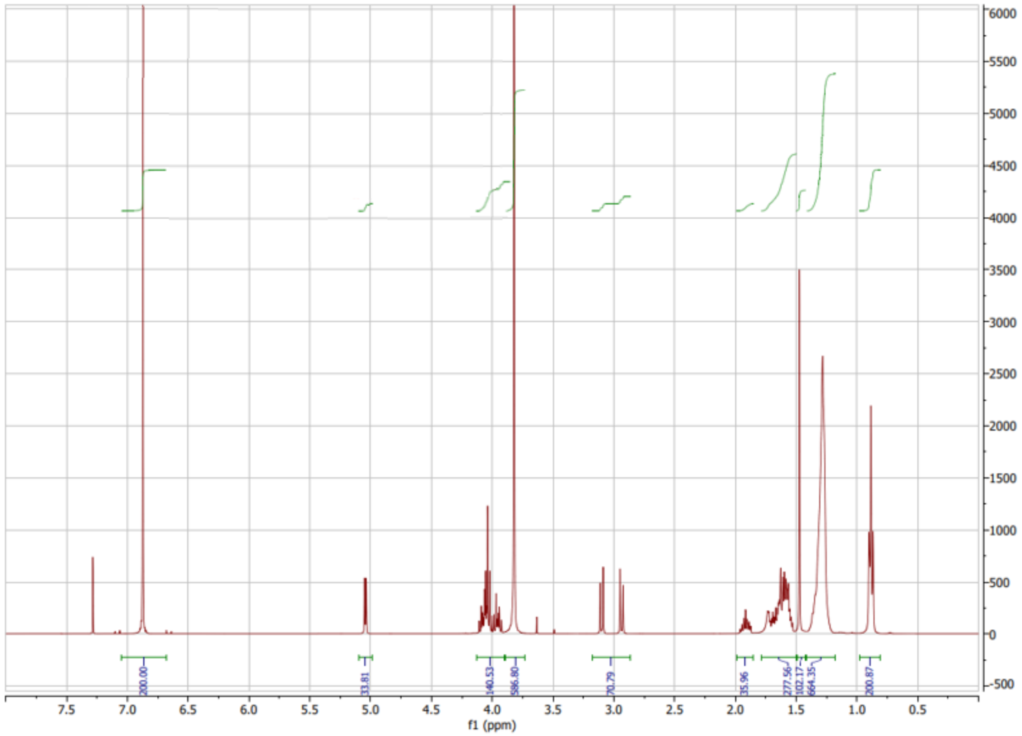

Figure S3. ^{1}H NMR spectra of MF/MA-C8 bio-plasticizer (with dimethyl fumarate as internal standard).

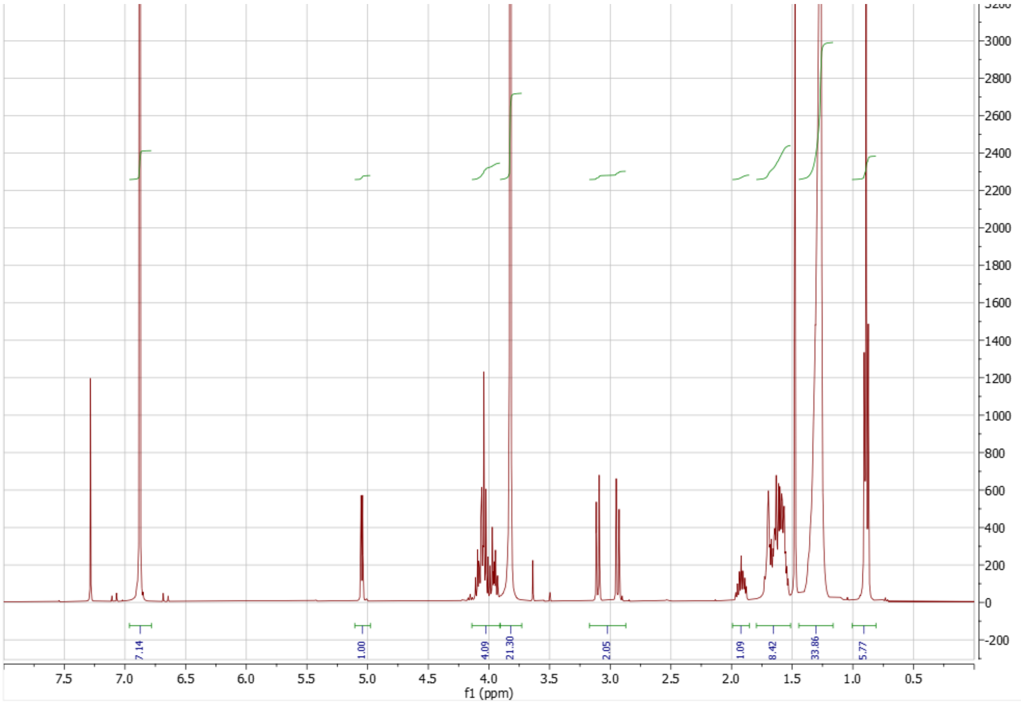

Figure S4. ^{1}H NMR spectra of MF/MA-C12 bio-plasticizer (with dimethyl fumarate as internal standard).

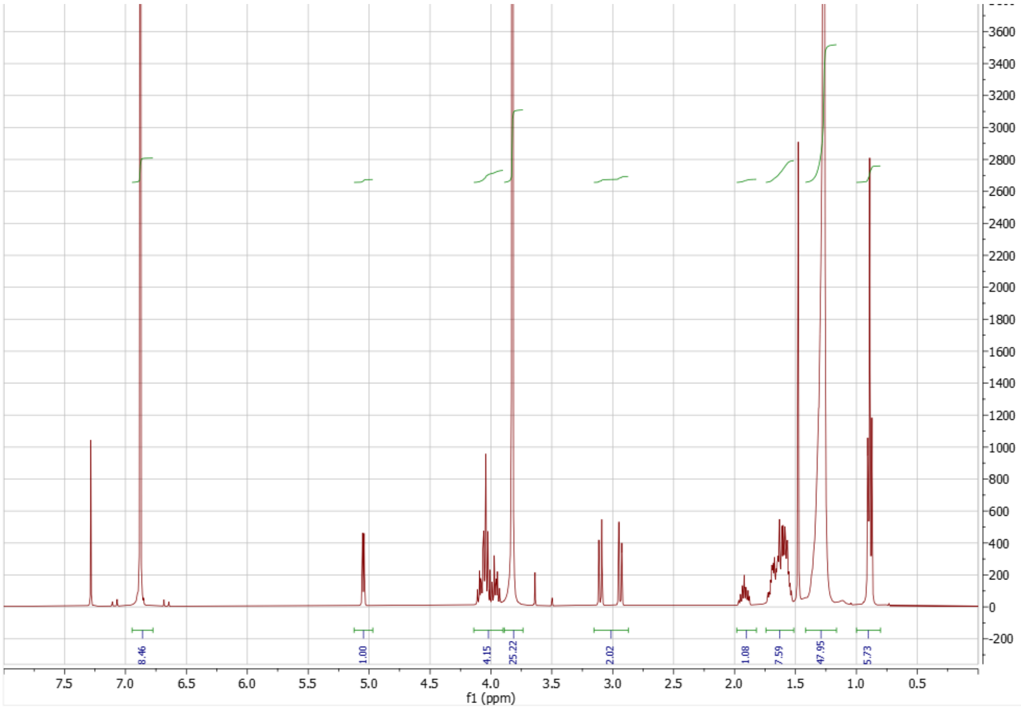

Figure S5. ^{1}H NMR spectra of MF/MA-C16 bio-plasticizer (with dimethyl fumarate as internal standard).

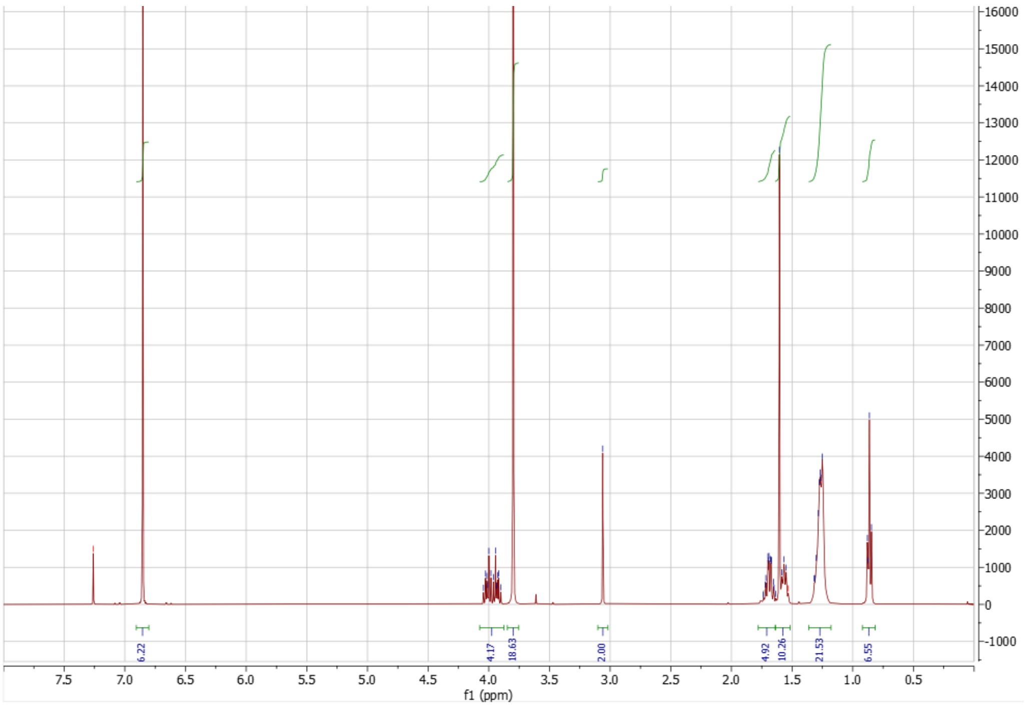

Figure S6. ^{1}H NMR spectra of DMF/MA-C8 bio-plasticizer (with dimethyl fumarate as internal standard).

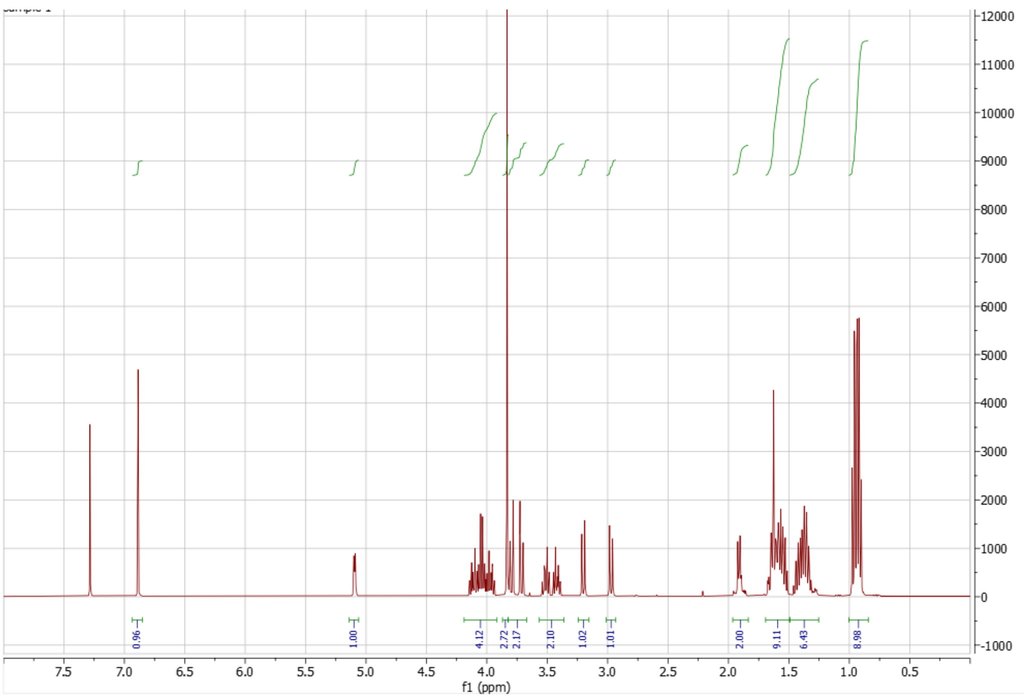

Figure S7. ^{1}H NMR spectra of 3-C4-DA bio-plasticizer (with dimethyl fumarate as internal standard).

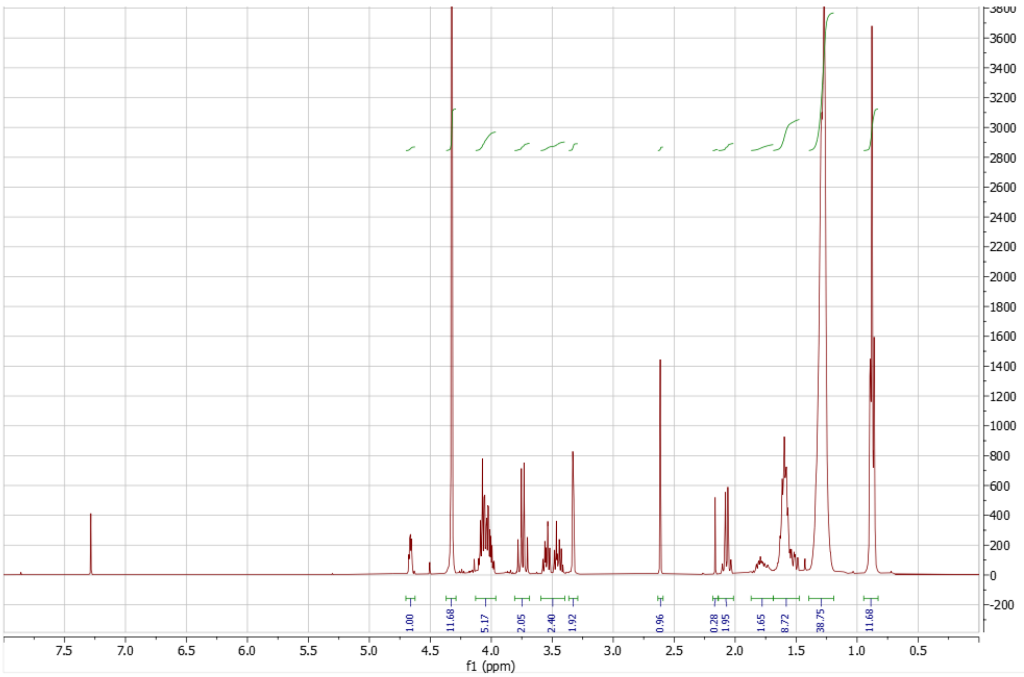

Figure S8. ^{1}H NMR spectra of 3-C8-DA bio-plasticizer (with nitromethane as internal standard).

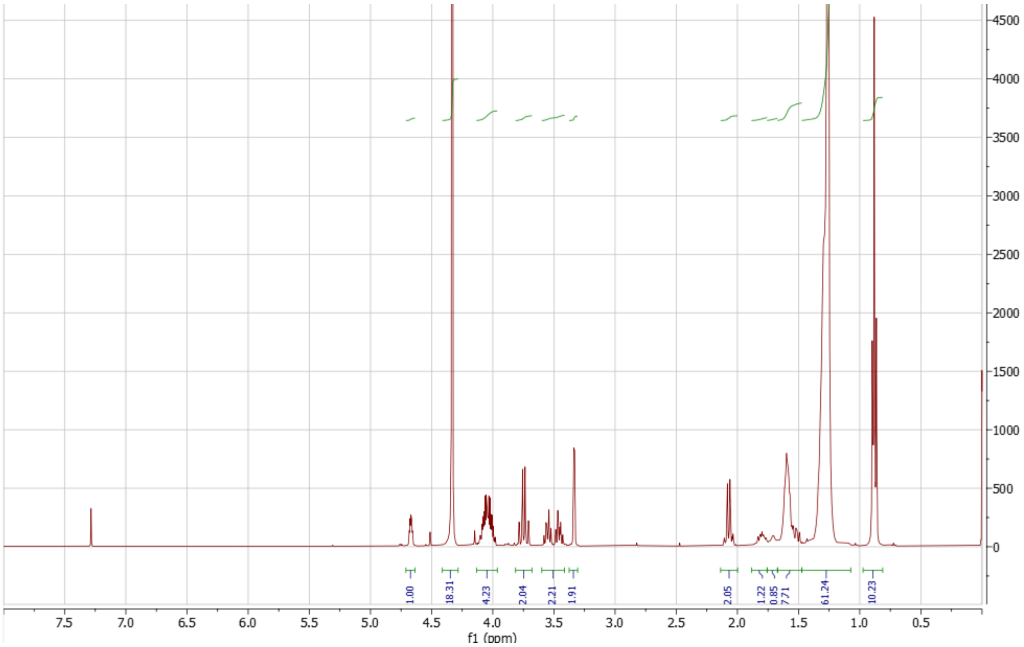

Figure S9. ^{1}H NMR spectra of 3-C12-DA bio-plasticizer (with nitromethane as internal standard).

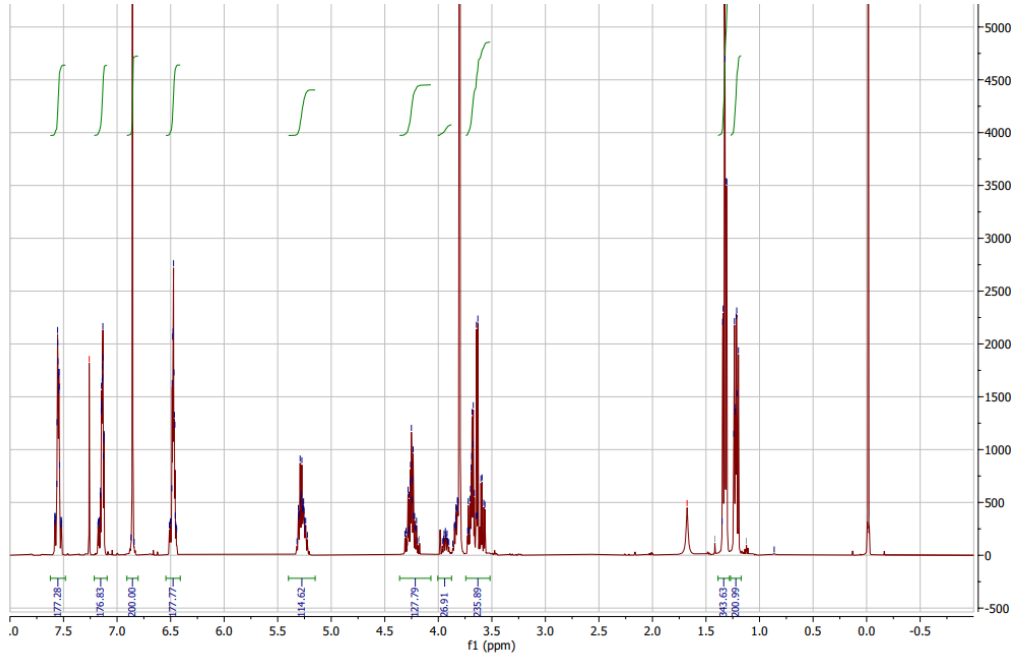

Figure S10. ^{1}H NMR spectra of Bis-F bio-plasticizer (with dimethyl fumarate as internal standard).

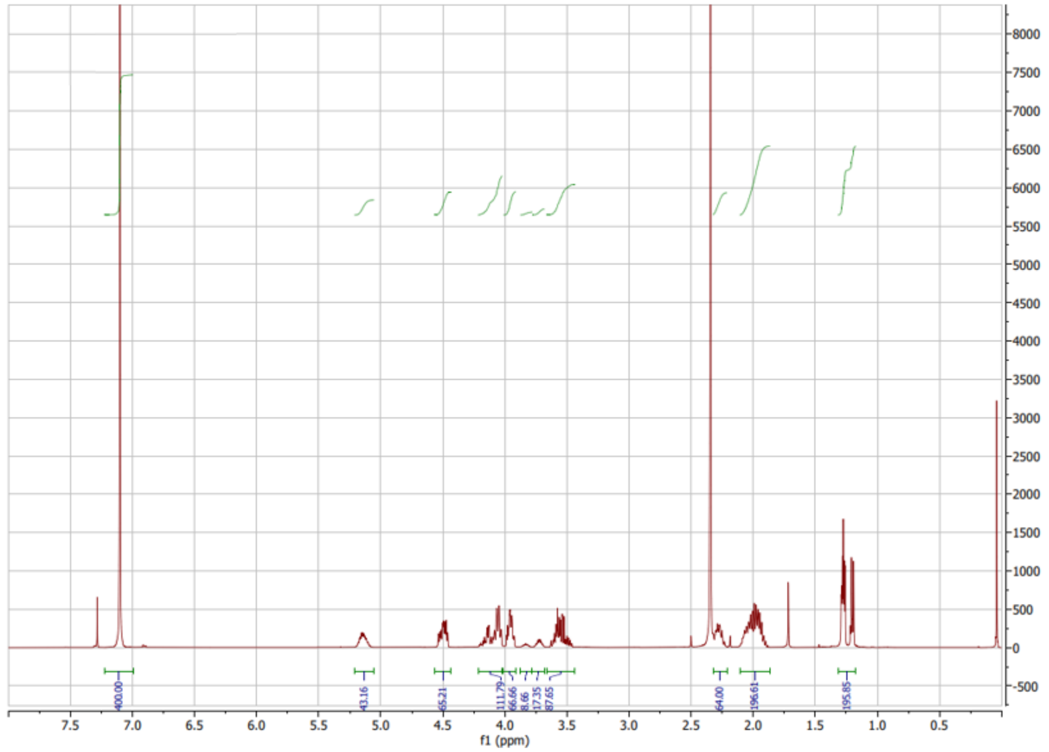

Figure S11. ^{1}H NMR spectra of Bis-THF bio-plasticizer (with p-xylene as internal standard).

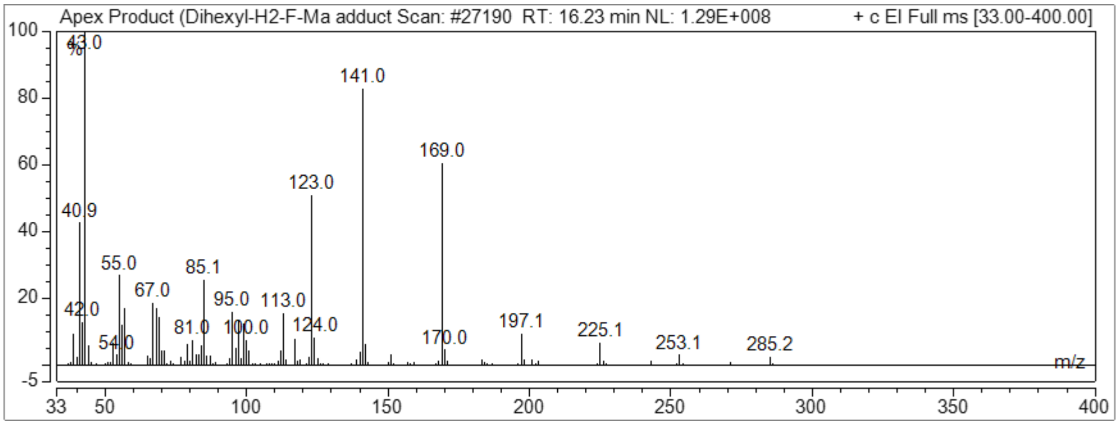

Figure S12. MS spectra of F/MA-C6 bio-plasticizer (m/z 33-400).

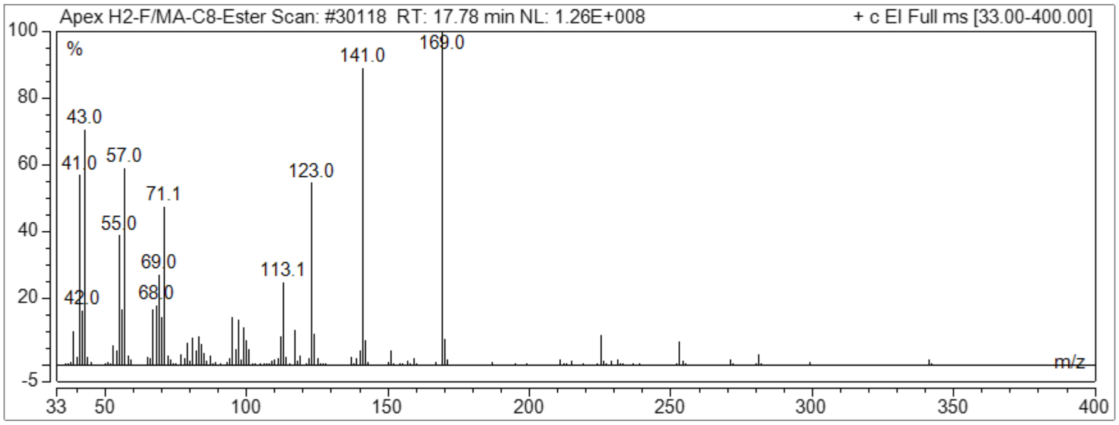

Figure S13. MS spectra of F/MA-C8 bio-plasticizer (m/z 33-400).

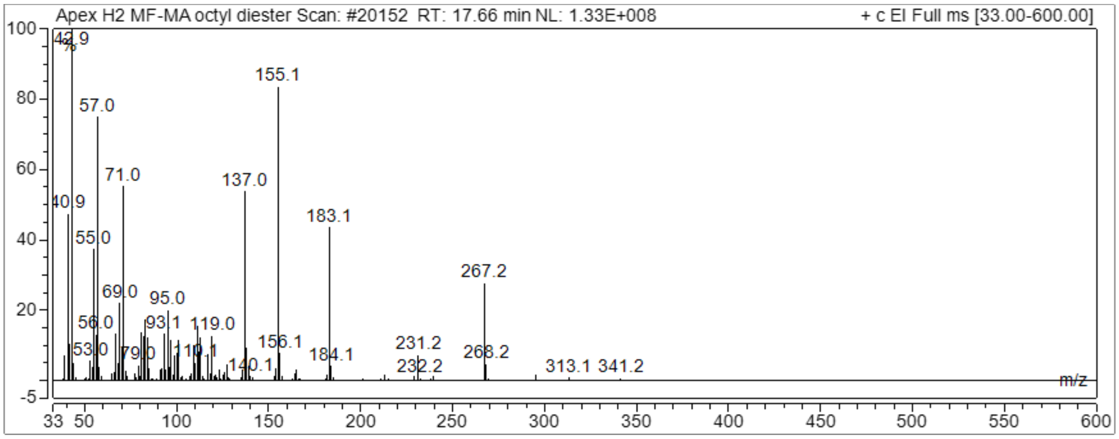

Figure S14. MS spectra of MF/MA-C8 bio-plasticizer (m/z 33-600).

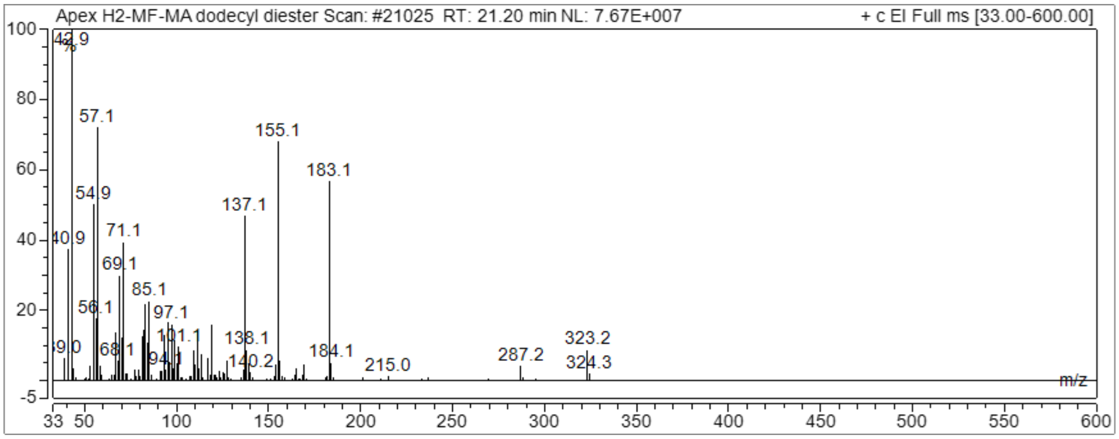

Figure S15. MS spectra of MF/MA-C12 bio-plasticizer (m/z 33-600).

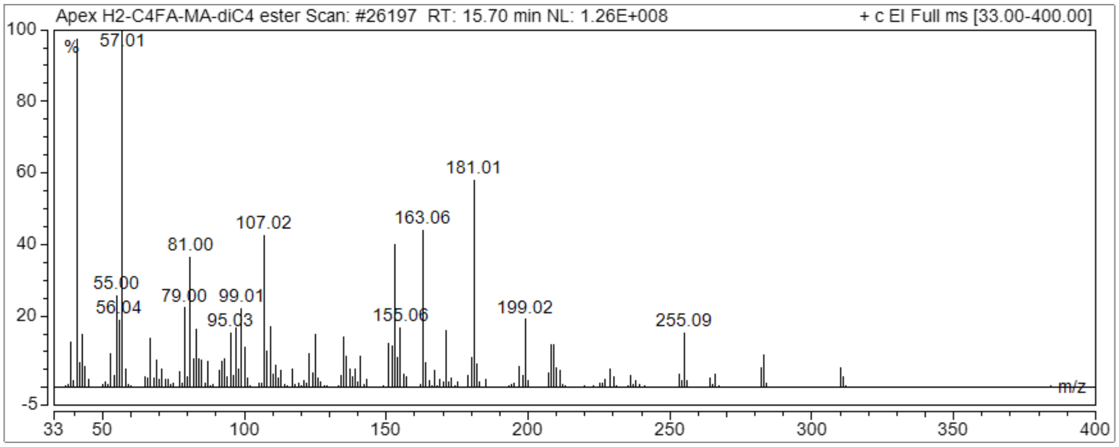

Figure S16. MS spectra of 3-C4-DA

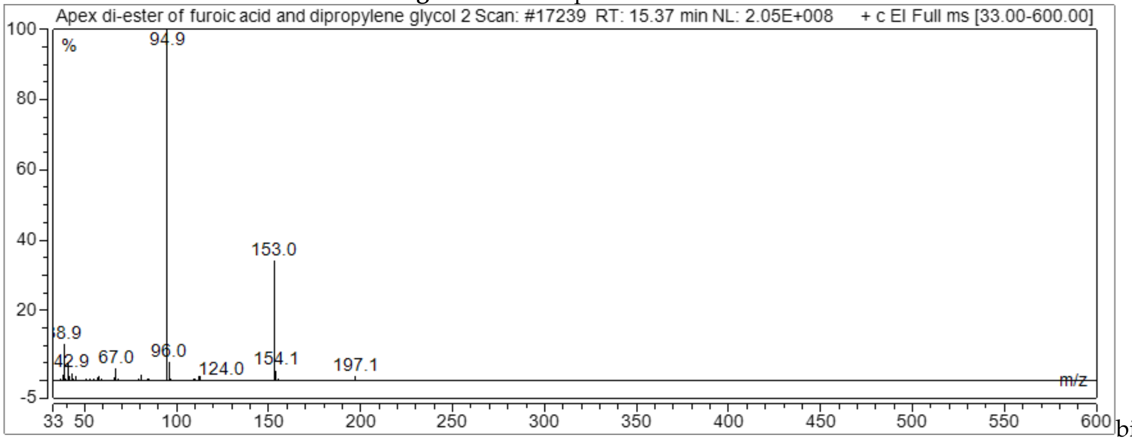

bio-
plasticizer (m/z 33-400).

Figure S17. MS spectra of Bis-F bio-plasticizer (m/z 33-600).

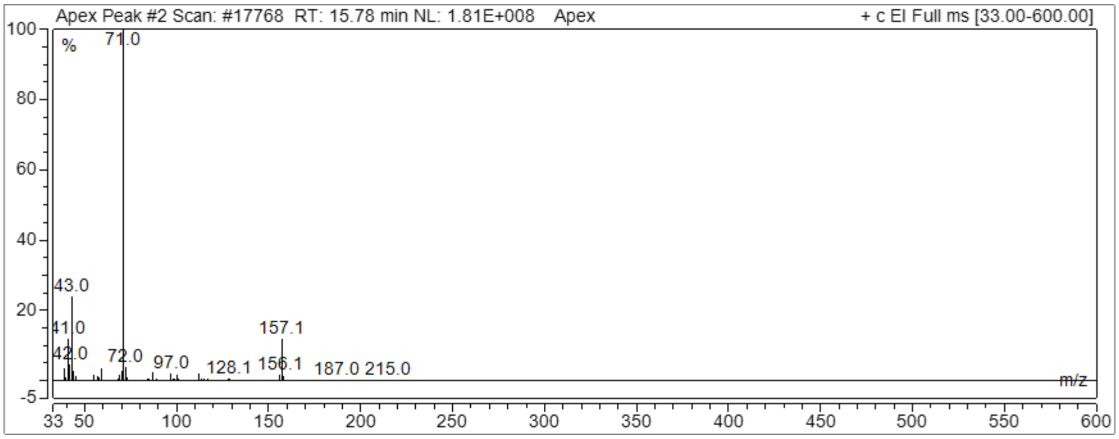

Figure S18. MS spectra of Bis-THF bio-plasticizer (m/z 33-600).

Table S1. Comparison of thermal degradation parameters (T_d-onset vs. T_d-max).

	Td-onset (°C)	Td-max (°C)	Difference (°C)	Difference (%)
**DINP**	295.1	331.5	36.4	12.3
**PEG-400**	263.7	361.3	97.7	37.0
**DPGDB**	269.5	310.9	41.5	15.4
**F-MA-C6**	226.4	258.1	31.6	14.0
**F-MA-C8**	252.4	282.8	30.5	12.1
**MF/MA-C8**	251.6	288.3	36.6	14.6
**MF/MA-C12**	316.3	355.4	39.1	12.3
**MF/MA-C16**	342.0	377.9	35.9	10.5
**DMF-MA-C8**	282.4	317.6	35.2	12.5
**3-C4-DA**	244.6	281.4	36.9	15.1
**3-C8-DA**	301.3	347.3	46.1	15.3
**3-C12-DA**	362.7	394.8	32.2	8.9
**Bis-F**	248.8	283.6	34.8	14.0
**Bis-THF**	247.9	287.6	39.7	16.0

Table S2. Hansen Solubility Parameters of (bio)-plasticizers and polymers (PVC and PLA).

	δD	δP	δH
**DEHP**	16.0	4.9	2.3
**DINP**	16.3	4.9	2.3
**PEG-400**	15.7	7.5	9.7
**DPGDB**	18.0	6.2	3.7
**F/MA-C6**	16.6	3.7	4.2
**F/MA-C8**	16.5	3.0	3.4
**MF/MA-C8**	16.7	2.9	3.1
**MF/MA-C12**	16.5	2.3	2.4
**MF/MA-C16**	16.5	1.9	1.9
**DMF/MA-C8**	16.8	2.8	2.8
**3-C4-DA**	16.7	4.1	3.6
**3-C8-DA**	16.6	3.0	2.4
**3-C12-DA**	16.6	2.3	1.7
**Bis-F**	17.8	8.5	5.2
**Bis-THF**	17.0	7.6	6.4
**PVC**	18.8	9.2	6.3
**PLA**	18.5	8.0	7.0
